# Characterizing Evoked Movement Patterns and Connectivity in Sensorimotor Cortex After Sensory Loss in Squirrel Monkeys

**DOI:** 10.1002/cne.70099

**Published:** 2025-10-17

**Authors:** Hui‐Xin Qi, Chia‐Chi Liao, Jamie L. Reed, Iwona Stepniewska, Qimeng Wang, Jon H. Kaas

**Affiliations:** ^1^ Department of Psychology Vanderbilt University Nashville Tennessee USA

**Keywords:** cortical connections, long‐train intracortical microstimulation, mapping, plasticity, primate, spinal cord injury

## Abstract

Sensory feedback is crucial for movement execution, especially in the highly specialized skilled hand use in humans and other primates. Extensive sensory loss of tactile and proprioceptive inputs from the hand initially results in severe motor deficits, but recovery occurs gradually over time. To determine how sensory loss from one hand affects evoked forelimb movements from sensorimotor cortical areas, lesions were selectively placed in the ascending somatosensory pathway in the dorsal column in the cervical spinal cord (C5, DCL) of three squirrel monkeys. After 1 year of postlesion recovery, we examined the long‐term effects of DCL on motor response patterns evoked by long‐train intracortical microstimulation (LT‐ICMS) from the hand cortex in primary motor cortex (M1) and somatosensory areas 3a, 3b, and 1 in both control and DCL groups. Somatosensory receptive fields were mapped primarily to define the borders of area 3b, as well as to compare somatosensory responses with motor responses after DCL. Corticocortical connections were investigated by injecting neuroanatomical tracers into the sensory‐deprived forelimb regions of 3b and M1. We found that after somatosensory loss and compensation, LT‐ICMS‐evoked movement maps for M1 and deprived somatosensory areas did not show statistically detectable effects of DCL. Results were consistent with our earlier findings of the effects of long‐term recovery from DCL on somatosensory response maps and connections among somatosensory areas. We now show that the effects of long‐term DCL on evoked motor responses in somatosensory cortex were limited, while the connections between M1 cortex and somatosensory cortical areas were more widespread.

## Graphical abstract

Excitability to long‐train intracortical microstimulation in the primary somatosensory cortex after recovery from long‐term sensory loss tended to be altered but was not statistically different from that of normal and intact hemispheres. Cortical connection patterns of areas 3b and M1 were consistent with early findings, characterized by the presence of sparse but widespread connections after sensory loss. The results suggest that the sensorimotor system undergoes both anatomical and functional reorganization in response to compensating for sensory loss.

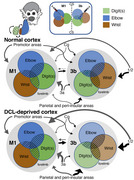

## 1 Introduction

Extensive sensory loss of tactile and proprioceptive inputs from the forelimb in monkeys results in both sensory and motor impairments (Florence et al. 2000; Darian‐Smith and Ciferri 2005; Qi et al. 2013; Duque et al. 2023). We reasoned that motor maps of evoked movements that exist in the somatosensory cortex are likely present in all primates, such as in prosimian galagos (Fogassi et al. 1994), marmosets (Burish et al. 2008), and macaque monkeys (Baldwin et al. 2018; Bresee et al. 2024). And if so, we asked, if lesions of the somatosensory pathway impact the motor maps in a similar way in both the somatosensory cortex and motor cortex? Here, we determined if the somatosensory cortex of squirrel monkeys has a motor map of the forelimb that correlates with the responsiveness of the sensory map without and after recovery from sensory loss. As sensory loss results in changes in cortical connections (Florence et al. 1998; Liao et al. 2016a), we also labeled the connections between the maps in the somatosensory and motor cortex. We also considered possible changes in the motor map in the motor cortex and in the connections between the somatosensory and motor cortex.

Our study objective was to determine if the organization of evoked motor response patterns in the forelimb region of the sensorimotor cortex is affected by long‐term sensory loss and if cortical connections are likely to provide critical substrates for functional recovery. We specifically examined the anatomical and functional organizations in the primary motor cortex (M1; for other abbreviations, see Table 1) and somatosensory cortex areas 3a, 3b, 1 in squirrel monkeys. Squirrel monkeys provide the advantage in such experiments of having the hand and forelimb representations of the sensorimotor cortex exposed on the brain surface for visually directing the placement of the electrodes for functional mapping and placement of injections for cortical neuron tracing.

**TABLE 1:** Abbreviations.

1	Area 1
2	Area 2
3a	Area 3a
3b	Area 3b/primary somatosensory cortex
BDA	Biotinylated dextran amine
Cg	Cingulate gyrus
CgS	Cingulate sulcus
CO	Cytochrome oxidase
CTB	Cholera toxin subunit B
CS	CS central sulcus
DC	Dorsal column
DCL	Dorsal column lesion
FR	Fluororuby
M–W	Mann–Whiney *U*‐test
K–S	Kolmogorov–Smirnov test
K–W	Kruskal–Wallis test
LT‐ICMS	Long‐train intracortical microstimulation
M1	Primary motor cortex
PMd	Dorsal premotor cortex
PMc	Ventral premotor cortex
PPC	Posterior parietal cortex
PR	Parietal rostral area
PV	Parietal ventral somatosensory area
Ri	Retroinsular cortex
S1	Primary somatosensory cortex (area 3b)
S2	Secondary somatosensory area
SMA	Supplementary motor area
ST‐ICMS	Short‐train intracortical microstimulation
VP	Ventroposterior nucleus of the thalamus
VGLUT2	Vesicular glutamate transporter type 2
VPLo	Oral part of ventroposterior lateral nucleus of thalamus
VS	Ventral somatosensory area

Extensive lesions of the cuneate fasciculus of the dorsal columns (DCs) at a mid‐cervical level of the spinal cord lead to a loss of most of the direct somatosensory afferent inputs to the cuneate nucleus and result in impaired dexterity of the ipsilateral hand (Qi et al. 2013, 2021). The input‐deprived forelimb region of area 3b is completely unresponsive to touch on the affected hand and arm immediately after the lesions, but considerable reactivation of the forelimb representation in area 3b returns over months of recovery (Jain et al. 2008; Chen et al. 2012; Qi et al. 2016). The reactivation pattern, while clearly abnormal, is enough to allow skilled hand use to reappear. The reactivation depends on the potentiation of a small number of preserved DC axons from the hand and possibly the potentiation of a larger number of second‐order spinal cord neuron projections to the cuneate nucleus (Liao et al. 2015, 2018).

The mutability of the somatosensory cortex and its relationship to skilled hand use suggest that sensory deprivations might change motor response function as well in the sensory‐deprived cortex. Early studies emphasized the findings that somatosensory areas of the cortex have a motor component, and motor areas have a sensory component (see Harlow and Woolsey 1958). Early studies also found that cortical motor maps are mutable in response to skilled learning and altered sensory experiences due to nervous system injuries (see Mohammed and Hollis 2018 for review).

Motor responses from the motor cortex have been studied extensively in various species of mammals, including nonhuman primates and humans (for reviews, see Schieber 2001; Capaday et al. 2013; Lemon and Morecraft 2023). In squirrel monkeys, forelimb response territories of M1 do not have a precise somatotopic organization, and cortical locations that activate different muscles show considerable individual variation (Donoghue et al. 1992; Nudo et al. 1992). A more limited number of studies have examined evoked movements from the somatosensory cortex, as somatosensory cortex is less responsive to the commonly used short trains of electrical pulses (ST‐ICMS; Wannier et al. 1991), particularly under anesthesia (e.g., Wu and Kaas 1999; Burish et al. 2008). Longer trains of electrical pulses (50 ms vs. 500 ms) do not affect the evoked muscle activation pattern, but changes in current intensity can (Capaday 2022). Longer pulse sequences evoke purposeful movements (Graziano et al. 2002a; Taylor and Gross 2003), and such long‐train intracortical microstimulation (LT‐ICMS; see Graziano et al. 2002b for review) has enabled researchers to more effectively investigate movement organization in the somatosensory cortex of anesthetized animals, including rodents (Halley et al. 2020), tree shrews (Baldwin et al. 2017), and macaque monkeys (Baldwin et al. 2018). However, LT‐ICMS‐evoked motor response patterns from the somatosensory cortex of squirrel monkeys have not been systematically investigated previously. Furthermore, the question of whether motor response patterns in the somatosensory cortex are affected by sensory loss from the hand has not been studied in primates, and it was not clear if loss if sensory loss has long‐term effects along with the effects on connections of motor cortex with somatosensory areas. Both normal and postlesion maps and connection patterns are important to report.

To this end, we address three questions. First, what are the organizations of the motor maps in the somatosensory cortex with intact somatosensory organization? Second, are the LT‐ICMS excitabilities of the hand and forelimb representations of sensorimotor areas M1, 3a, 3b, and 1 altered after 1 year of recovery from lesions of the DC somatosensory pathway in the spinal cord? Third, could changes in cortical connections of sensorimotor areas alter cortical motor response map organization after long‐term sensory loss from dorsal column lesions (DCLs)? We found anatomical and functional alterations after sensory loss that reflected close relationships between the somatosensory and motor systems, emphasizing the usefulness of considering them as parts of a sensorimotor system.

## 2 Materials and Methods

Four adult male squirrel monkeys (*Saimiri boliviensis*) were examined in this study. Three squirrel monkeys received a unilateral DCL at the cervical C5 level of the spinal cord. Data were compared to the results obtained from one normal monkey, for which the forelimb maps were similar to those published in other studies (e.g., Sur et al. 1982; Merzenich et al. 1983). We identified the representations of fine forelimb movements in the hand regions of areas 3b, 3a, and 1, and the forelimb region of M1 contralateral to the lesion side after a year of recovery from somatosensory input deprivation from the hand. In two of the three lesioned monkeys, the sensorimotor cortex ipsilateral to the DCLs was also mapped. In the normal monkey, the left and right sensorimotor cortices were mapped. To reveal the sensorimotor interactions at the cortical level, two distinguishable anatomical tracers were injected into the territories where similar movements were elicited in both 3b and M1 (matching), or where different movements were elicited in 3b and M1 (nonmatching). The distributions of labeled neurons were aligned with a movement representation map (motor map). All surgical procedures and animal care followed the guidelines of the National Institutes of Health *Guide for the Care and Use of Laboratory Animals*. They were approved by the Animal Care and Use Committee of Vanderbilt University.

### 2.1 Surgical Procedures

The general surgical procedures were as described in early studies (e.g., Qi et al. 2011a; Liao et al. 2016a). In brief, monkeys were initially tranquilized with an intramuscular injection of ketamine hydrochloride (10–25 mg/kg), and anesthesia was continued by inhaled isoflurane (1%–2% mixed in O_2_) during the preparation and surgical procedures. While fully anesthetized, the monkey was intubated and cannulated. After the head was secured in a stereotaxic frame, vital signs, including heart rate, respiration rate, body temperature, exhaled CO_2_, and arterial O_2_ saturation, were monitored every 10 min throughout the surgery to maintain a consistent level of anesthesia. During the electrophysiological recording for somatosensory mapping and microstimulation for motor mapping, the anesthesia was maintained by an intravenous administration of ketamine hydrochloride (12 mg/kg/h). Supplemental xylazine (0.4 mg/kg, IM) was given when needed to suppress spontaneous movements. These procedures were performed under aseptic conditions, and each monkey was closely monitored during recovery from the anesthesia. Antibiotics were provided perioperatively, and analgesics were given every 12–24 h postoperatively for 3 consecutive days.

#### 2.1.1 Dorsal Column Lesions

In three squirrel monkeys (SM‐C, SM‐J, and SM‐S), the cuneate fasciculus of the DC on the right side of the spinal cord was cut at the C5 level to spare some of the inputs from digit 1 and possibly a few from digit 2 (Florence et al. 1991; Qi et al. 2011a). Details of the lesion methods have been described elsewhere (Jain et al. 1997; Qi et al. 2011a; Liao et al. 2016a). In brief, aseptic surgery for laminectomy of the cervical C4 and C5 vertebrae was performed, and the underlying dura covering the spinal cord was opened. The locations of the representations of digits 1 and 2 in the dorsal horn of the spinal cord were identified by electrophysiological recordings (somatosensory mapping). After part of the hand representation was defined in the dorsal horn of cervical C5, we used fine forceps to clamp the DC between the representations of digits 1 and 2 on the right side for 2 min, followed by a cut with iris surgical scissors at the same location (Liao et al. 2016a).

### 2.2 Microelectrode Recording for Somatosensory Mapping

The organizations of the hand regions of somatosensory areas 3b, 3a, and 1 were revealed by microelectrode recording in one normal monkey (SM‐R) and three monkeys after 11–13 months of recovery from DCL (SM‐C, SM‐J, SM‐S, Table 2). In all monkeys, the left hemisphere sensorimotor cortex (contralateral to the spinal cord lesion site) was carefully and thoroughly mapped, while a small part of the right somatosensory cortex (ipsilateral to the lesion) was also mapped in SM‐R, SM‐C, and SM‐S to reveal the borders of the area 3b hand region. Details of the somatosensory mapping methods have been described in many reports (e.g., Merzenich et al. 1978; Sur et al. 1982; Jain et al. 1997; Florence et al. 1998; Qi et al. 2011a). In brief, under general anesthesia with isoflurane (1%–2% in oxygen), a craniotomy was made over the frontal and parietal cortex of both cerebral hemispheres to expose the forelimb and parts of the face regions in areas 3b, 3a, 1, 2, and M1. The exposed dura covering the brain was retracted to allow access to microelectrode penetrations. Recordings were performed with low‐impedance tungsten microelectrodes (1 MΩ at 1 kHz) that were lowered perpendicularly through the cortical surface to the depth of 650 µm where cortical layer 4 was located. We systematically placed the microelectrode every 350–500 µm to examine the neuronal receptive fields across the hand regions of areas 3b, 3a, 1, and 2, which are medially bordered by the representation of wrist and forearm, and laterally neighbored by the representation of face. Sites where no receptive field could be identified were marked as “no response.” The rostral and caudal boundaries of area 3b were identified based on the reversed somatotopies of posteriorly adjacent area 1, and differences in the response properties of the three fields. The locations of recording sites relative to the vascular distribution were marked on a printed photo of the exposed brain surface, and the locations of the corresponding receptive fields were later used for the reconstruction of somatosensory maps. The responsiveness to tactile stimulation in the forelimb region of somatosensory area 3b was calculated by counting the total responsive sites to tactile stimulation divided by the total number of mapping sites in area 3b of each monkey, expressed as a percentage.

**TABLE 2:** Case information.

Case	Lesion level	Lesion extent	Recovery time (day/week)	Tracer (transport time)	Area of injection	Threshold movement at injection site	Figures
SM‐R	Normal	N/A	N/A	BDA/CTB *(15 days)*	3b	Forearm supination	1, 2, 5, 7, 11, 12
				FR *(15 days)*	M1	Digit flexion and forearm supination (grasp)	
SM‐C	rC5	98%	329/47	BDA/CTB *(27 days)*	3b	Forearm supination	1, 2, 3, 5, 8, 11, 12
				FR *(13 days)*	3a	Forearm supination	
SM‐J	cC5	95%	399/57	BDA/CTB *(35 days)*	3b	Digit 1	1, 3, 5, 9, 11, 12
				FR *(14 days)*	M1	Digit, wrist, elbow concurrent (grasp)	
SM‐S	midC5	90%	357/51	BDA/CTB *(27 days)*	3b (near 3a–3b border)	Digit, elbow, wrist	1, 2, 3, 5, 10, 11, 12
				FR *(13 days)*	M1	Digit flexion and forearm supination (grasp)	

Case information listing the level and extent of dorsal column lesion, recovery time, types of tracers, and transportation times, the location of tracer injections, and results that correlate with figures.

Abbreviations: cC5, caudal C5 of cervical spinal cord; midC5, middle C5 of cervical spinal cord; rC5, rostral C5 of cervical spinal cord. Other abbreviations are consistent with those in Figure 7.

### 2.3 Motor Mapping With Long‐Train Intracortical Microstimulation

Details of the microstimulation mapping methods have been described elsewhere (Stepniewska et al. 2005; Gharbawie et al. 2011). In brief, after somatosensory mapping, under ketamine anesthesia with supplemental xylazine, biphasic LT‐ICMS was delivered through low‐impedance microelectrodes (0.9–1.2 MΩ at 1 kHz; Microprobes, Inc.) to map sensorimotor areas 3a, 3b, 1, and M1. The electrical stimulus was generated using a Master 8 stimulator (A.M.P.I., Jerusalem, Israel) with a Biphasic Stimulus Isolator (Bak Electronics Inc., Umatilla, FL, USA). The stimuli consisted of a train of 150 biphasic pulses (500 ms duration at 300 Hz) with a pulse duration of 0.2 ms. The current intensity was initially set at 60 µA and was increased up to 150 µA as needed to evoke movement. A current intensity of 150 µA was considered safe, based on Graziano et al. (2002a). Penetration sites were marked on a high‐resolution digital photograph of the exposed cortex for reconstructing the motor maps. The current thresholds and types of evoked movements were documented. Sites where no movement was elicited at the current intensity of 150 µA were marked as “no response.” Threshold values were determined as the lowest current amplitude for an evoked movement to be detected. All movements were characterized by two independent observers and were video‐recorded for offline analysis and confirmation. We closely watched if there was any bilateral movement during the experiment, as well as offline video analysis.

### 2.4 Tracer Injections

#### 2.4.1 Cortical Injection Into Sites That Produce Movements

At the end of the initial recording and stimulation session, the aseptic conditions were continued using isoflurane anesthesia, and tracers were injected into selected locations in areas 3b and M1. Tracers were pressure injected with 1 µL or 2 µL Hamilton syringes outfitted with glass pipettes beveled to a sharp tip (25–50 µm diameter). Three tracers used in the experiments included biotinylated dextran amine (BDA; Invitrogen, Carlsbad, CA, USA; 10% in 0.1 M PB, 3k and 10k MW), cholera toxin subunit B (CTB; Sigma‐Aldrich, St. Louis, MO, USA; 1% in distilled water), and Fluororuby (FR; Invitrogen, 10% in 0.1 M phosphate buffer (PB), 3k and 10k MW). CTB (0.06 µL total) was injected into the representations of digit or elbow movements in area 3b. BDA (1 µL total) was injected into part of area 3b adjacent to the CTB injection site that had the same representation of a forelimb movement. In one case (SM‐J), a BDA and CTB mixture was injected into a single site in the digit movement representation in area 3b. FR (1 µL total) was injected into a matching or nonmatching representation of digit or elbow movements in M1. Injections were placed at three depths beneath the surface of the cortex (0.5, 1.0, and 1.5 mm) at each injection site. After the micropipette was withdrawn, the dura was repositioned over the cortex and the exposed surface was protected by a piece of Gelfilm (Pfizer) before closing the skull opening with dental cement. The skin was closed, and the monkey was monitored as it recovered from anesthesia, and analgesics were given for 3 consecutive days. Approximately 2–3 weeks were needed for tracer transportation before the start of terminal mapping procedures.

#### 2.4.2 Subcutaneous Digit Injection

We examined if digit 1 inputs above or around the DCL traveled in the spinal cord to the cuneate nucleus and whether the spared digit inputs expanded their terminal fields outside the expected representations in the cuneate nucleus. To help estimate the anatomical effects of the DCL, we injected a transganglionic transport tracer CTB conjugated to horseradish peroxidase (B‐HRP; 10–12 µL, 0.2% in dH_2_O, Invitrogen) into digit 1 of both hands. In the lesioned monkeys, injections were made after long‐term recoveries (11–13 months) from unilateral DCL. A terminal procedure was performed 5 days after tracer injections to allow transportation from the digits to the spinal cord and the cuneate nucleus (Qi et al. 2011a; Liao et al. 2015). In one lesioned monkey (SM‐J), B‐HRP was injected into the distal phalanges of digits 1 (12 µL), 3 (5 µL), and 5 (5 µL) of the left (the intact) hand, and a matching site on digit 1 (12 µL) of the right hand that was ipsilateral to the spinal cord DCL. The percentage of spared inputs to the cuneate nucleus was later estimated as the difference in cuneate nucleus labeling by digit 1 injections between the intact and lesioned sides (see Data analysis section).

### 2.5 Perfusion and Histology

At the end of the microelectrode recording and stimulation sessions, the mediolateral, rostral, and caudal borders of the hand representation region of area 3b were identified. The locations were marked with small electrolytic lesions made with a continuous current at 10 µA while the microelectrode was slowly withdrawn from a depth of 2 mm to the brain surface. Each monkey was then euthanized with an injection of sodium pentobarbital (120 mg/kg, IV). After confirmation of death, transcardial perfusion was made through the ascending aorta with 0.1 M phosphate‐buffered saline, PBS), followed by 3% paraformaldehyde in 0.1 M PB and subsequently 10% sucrose‐containing fixative. The brain and spinal cord were removed, immersed in 30% sucrose in PB for cryoprotection, and refrigerated overnight. In all monkeys, both hemispheres of the cortex were separated from the subcortical structures and flattened (see details in Gharbawie et al. 2011). Then after 24 h, cortices were cut parallel to the brain surface at a thickness of 40 µm, and sections were divided into three series to reveal fluorescent (FR) label, BDA and CTB, and for architectural structures with immunoreaction against vesicular glutamate transporter 2 (VGLUT2) antibodies to reveal rostral and caudal borders of area 3b (Liao et al. 2016b). The brainstem was cut in the transverse plane at a thickness of 50 µm. Sections of the brainstem were divided into series for revealing FR labels, BDA and CTB, and B‐HRP labeling (Qi and Kaas 2006), and for architectural structures with cytochrome oxidase (CO; Wong‐Riley 1979) and VGLUT2 expression (Liao et al. 2016b). To preserve information on the spinal cord segments during the histology processing, we first marked each spinal segment's boundaries with pins based on identifying the rostrocaudally arranged dorsal roots prior to tissue sectioning. The cervical spinal cords were cut at 50 µm in the transverse plane (SM‐J) or at 40 µm in the horizontal plane (SM‐R, SM‐C, SM‐S). The horizontally cut spinal cord sections were divided into three series for visualizing FR, BDA and CTB, and B‐HRP labeling. The transverse‐cut spinal cord sections were divided into four series to reveal FR labeling, BDA and CTB and B‐HRP labeling, and architectural structures. For the B‐HRP series, all sections through the brainstem and spinal cord were processed with tetramethyl benzidine (TMB) as a chromogen (Gibson et al. 1984).

#### 2.5.1 Antibody Characterization

Anti‐VGLUT2 primary antibody (RRID: AB_2187552): Mouse anti‐VGLUT2 recombinant protein (monoclonal), Millipore Catalog no. MAB5504, Burlington, MA, USA. The immunogen is a KLH‐conjugated linear peptide. In Western blots of primate neocortex, the antibody recognizes a 56‐kDA band, the molecular weight of VGLUT2 (Baldwin et al. 2013). This primary antibody was used in a concentration of 1:5000. Also see Turner et al. (2020).

#### 2.5.2 Immunohistochemistry

A mouse monoclonal anti‐VGLUT2 antibody was used (MAB5504, Millipore, Billerica, MA, USA). Previous reports indicate that VGLUT2 immunostaining reveals the thalamocortical terminations in layer 4 of the cortex in rats (Fujiyama et al. 2001), ferrets (Nahmani and Erisir 2005), galagos (Wong and Kaas 2010), and primates (Hackett and de la Mothe 2009; Balaram et al. 2013; Balaram and Kaas 2014; Baldwin et al. 2013; Liao et al. 2015), as well as the brainstem terminations in the thalamus of rats (Kaneko and Fujiyama 2002), squirrels (Wong and Kaas 2008), and primates (Qi et al. 2011b; Liao et al. 2016b; Takahata et al. 2018). In brief, sections were incubated in a blocker of 0.1 M PBS, pH 7.2, with 0.5% Triton X‐100 and 5% normal horse serum for 1 h at room temperature before incubation in primary antibodies (mouse anti‐VGLUT2, 1:5000, Millipore) in the blocker for 40–48 h at 4°C. After rinsing, the sections were incubated in the blocker containing biotinylated horse anti‐mouse IgG (Vector Laboratories, Newark, CA, USA; 1:200) for 90 min at room temperature followed by avidin/biotin‐based peroxidase “ABC” incubation (one drop each of reagent A and B per 7 mL of 0.1 M PB, pH 7.2; ABC kits, PK‐6100, Vector) for 90 min at room temperature. Immunoreactivity was visualized by developing sections with diaminobenzidine histochemistry with 0.03% nickel ammonium sulfate.

To visualize labeling from BDA and CTB injections (see details in Liao et al. 2013), we used a staining protocol (Angelucci et al. 1996) that revealed both the CTB and BDA labeling. In brief, sections were incubated in a blocker of 0.05 M TBS, pH 7.2, with 0.5% Triton X‐100 and 5% normal rabbit serum for 1 h at room temperature before incubation in primary antibodies (goat anti‐CTB, 1:5000, List Biological) in the blocker for four nights at 4°C. After rinsing, sections were incubated in the secondary antibody solution (anti‐goat IgG 1:200, PK‐4005; Vector) for 1 h and in the ABC solution for 1 h. Finally, sections were reacted in the peroxidase substrate solution (SK‐ 4600; Vector) to show BDA‐ and CTB‐labeled neurons and axon terminal fields.

### 2.6 Data Analysis

#### 2.6.1 Evaluation of the Lesion Extent and Level

In all lesioned monkeys, the level of the DCL was identified based on pin holes marking cervical segments. To illustrate the lesion site in the spinal cord, we reconstructed lesions from a series of horizontally cut spinal cord sections for monkeys SM‐C and SM‐S. Images of the spinal cord sections were acquired as follows. Spinal cord images were aligned to a pinhole along the midlines of a series of sections from dorsal to ventral. The maximal extent of the lesion (missing tissue) and surrounding scar tissue, the white matter, and the gray matter were measured, and a transverse view of the lesion site was reconstructed using Adobe Illustrator software (Adobe Systems, San Jose, CA, USA).

To further evaluate the extent of DCL in the three lesioned monkeys, B‐HRP was injected into digit 1 of both hands 4–7 days before the terminal experiment. These injections labeled the corresponding representation of digit 1 in the cuneate nucleus. That differential labeling based on the target territory in the cuneate nucleus ipsilateral or contralateral to the DCL reflected the extent of the digit 1 fibers spared from the lesion. Here, we focused on the sizes of the patches of terminals labeled in the cuneate nucleus. Images of brainstem sections containing B‐HRP labels were acquired using a darkfield Nikon E800 microscope (Nikon Inc., Melville, NY, USA) and a Nikon DXM1200 camera. Each image was then uploaded to NIH ImageJ software, and areal sizes of B‐HRP labels were obtained from the left and right cuneate nucleus throughout their rostrocaudal extent. This allowed us to calculate the ratio of spared axon terminal fields to those of the intact side (see Qi et al. 2011a for details).

Thus, we evaluated lesion extent in two ways: the reconstruction of the location and extent of the lesion in histologically processed sections of the spinal cord, and the quantification of the difference in the amount of labeled axon terminals between the deprived and intact cuneate nucleus of the brainstem after injecting B‐HRP tracer into digits of both hands (Qi et al. 2011a).

#### 2.6.2 Reconstruction of Somatosensory and Motor Maps

We scanned the printed photograph of the exposed brain surface that was marked with microelectrode penetrations and imported it into Adobe Illustrator 2021. To reconstruct the somatosensory maps, we plotted the recording sites in areas 3b, 3a, and 1 based on the receptive fields and neural responses defined during the electrophysiological recordings. The neural responsiveness of each recording site was marked with an assigned symbol, and the representations of digits, palm pads, and face were contoured with green, gray, and yellow colors. The representations of digits 1–5 were further colored with five dark to light shades. The boundaries of the hand region of area 3b were carefully defined based on neuronal receptive field properties, responsiveness, and somatotopies (e.g., Sur et al. 1982).

To reconstruct the motor maps, we analyzed the movements from video frames captured during the experiment and documented the joints involved in the movements evoked at high (up to 150 µA) to low (at least 10 µA) current amplitudes at each stimulation site. The LT‐ICMS is effective in evoking a series of joint movements in the forelimb that can be classified into functional domains (e.g., hand‐to‐mouth, grasping, reaching, etc.; Stepniewska et al. 2014). As higher currents may activate more neurons (Sato and Tanji 1989; Strick 2002; Brock et al. 2013; Kumaravelu et al. 2022), we reconstructed the motor maps by using the movements evoked at the threshold current. The threshold current level at each site was marked using different sizes of dots to facilitate the comparison across cortical areas. The representations of movements of digits, wrist, elbow, and shoulder were contoured with green, brown, blue, and purple, respectively. The representations involving parts above the neck, including ear movements, eye blinks, or grimaces, were marked with yellow.

#### 2.6.3 Quantification of Excitability

To quantify responsiveness or excitability produced by electrical stimulation, we first calculated the percentage of responses for each cortical area using the following equation:

$$\%\mathrm{Response}=\frac{\mathrm{Number}\;\mathrm{of}\;\mathrm{Responsive}\;\mathrm{Sites}}{\mathrm{Number}\;\mathrm{of}\;\mathrm{Total}\;\mathrm{Sites}\;\mathrm{Tested}}\times 100\%$$

Second, for each cortical area, we calculated the ratios of sites categorized by specific forelimb movements (e.g., digit, wrist, elbow, shoulder, etc.) to total evoked movement sites, expressed as a percentage, using the following equation:

$$\%\mathrm{Movement}\;\mathrm{per}\;\mathrm{Category}=\frac{\mathrm{Movement}\;\mathrm{Category}\;\mathrm{Tally}}{\mathrm{Total}\;\mathrm{Responsive}\;\mathrm{Sites}}\times 100\%$$

The categories included digit(s) movements (D), which were characterized as including just single digit or multiple digit movements such as flexions, extensions, radial deviations, ulnar deviations, abduction, adduction, as well as sequential movements such as grasping; D+, including combinations of movements of digit(s) with proximal joints and/or muscles; A+, forelimb movement that was proximal to digit(s), including single joint movements or combined movements of wrist, elbow, shoulder; and F/N, including face, head/neck alone, or their combined movements. For some analyses, the categories D and D+ were combined.

#### 2.6.4 Determining Areal Boundaries of Sensorimotor Cortex

Sensorimotor areal boundaries can be better defined by combining electrophysiological mapping results with those from histological tissue processing. Electrolytic markers placed strategically at physiological borders enhance this approach. Rostral and caudal borders of area 3b were identified using mapping results consistent with previously established somatotopic maps in owl monkeys (Merzenich et al. 1978), squirrel monkeys (Sur et al. 1982), and titi monkeys (Coq et al. 2004). Typically, neurons in area 3b are robustly responsive to low‐threshold, cutaneous stimulation applied within their small and discrete receptive fields, whereas area 1 neurons have larger receptive fields that are responsive to light touch. The border between these two areas was more apparent in rows of recording sites that revealed the reversed somatotopies between areas 3b and 1. Area 3a neurons predominantly responded to high‐threshold tapping and muscle or joint manipulations (Sur et al. 1982). The border between areas 3a and M1 was estimated using LT‐ICMS current threshold values, as lower threshold movements (< 60 µA) were likely in M1 but not in 3a. The rostral and caudal borders of area 3b were also verified in adjacent sections processed for VGLUT2 immunostaining. The mediolateral hand–face borders of area 3a and M1 were determined by extending rostrally from the hand–face border of area 3b. Face regions of areas 3a, 3b, 1, and M1 were excluded from the calculation but were included in the reconstructed maps, as the study focused on the forelimb territory.

#### 2.6.5 Plotting of Labeled Neurons

The distributions of labeled neurons after BDA and CTB and FR injections in area 3b and M1 were systematically plotted using the Neurolucida system (MBF Bioscience, Williston, VT, USA). Locations of landmarks, vessels, and electrolytic lesions were carefully marked to facilitate the alignment of cortical sections from superficial to deep layers with the assistance of VGLUT2‐processed sections that revealed the extent of area 3b. To calculate and visualize the density of areal distribution of labeled neurons, heatmaps were created with a Python script (https://github.com/kaaslabqw/neuron‐density‐analysis, Wang et al. 2021).

#### 2.6.6 Alignment of Plotted Neurons and Motor Maps

To evaluate connectional densities of representations of movements of digits, wrist, elbow, and shoulder between areas 3b, 3a, and M1, we superimposed the plots of BDA‐ and CTB‐ and FR‐labeled neurons onto the LT‐ICMS‐defined motor maps based on the landmarks and lesion sites in four monkeys.

#### 2.6.7 Experimental Design and Statistical Analyses

For each electrode penetration in each case, the patterns of elicited movements (or lack of movement) and threshold information were entered into an Excel spreadsheet. Data were imported from Excel into statistical and graphics software (GraphPad Prism 9.0). We examined the similarity of somatosensory maps and motor response maps in area 3b within each mapped hemisphere by calculating the Spearman's correlation coefficient for the responsive proportion of mapped sites. This test was performed using the MATLAB “corr” command with the “type” argument “Spearman” and the “tail” argument “right” for the somatosensory responsiveness and motor responsiveness of area 3b from one hemisphere of each case. The next focus was on the characteristics of evoked motor response maps using summary values from each hemisphere as the subject unit, “n.” Kruskal–Wallis (K–W) nonparametric analysis of variance was used with Dunn's multiple comparisons test, for conditions divided by brain areas and movement representation types, regardless of DCL condition. This was performed for the proportion of distal movements to proximal movements, the proportion of responsive sites, and motor threshold values. To determine if motor threshold values significantly varied within individual subjects across cortical areas, we treated the areas within each hemisphere as repeated measures, regardless of DCL condition. Thus, we used the Friedman test with Dunn's multiple comparisons adjustment for this assessment with areas as the groups of interest and hemispheres as the subject units. When differences were not detected for subjects with and without DCL, we grouped the hemispheres based on the experimental variable of the known DCL condition for further analysis, and thus, intact hemispheres from DCL subjects were grouped with the normal hemispheres to form the control group. Statistical tests generally compared differences in motor response characteristics between the hemispheres directly affected by lesions and the hemispheres without direct sensory loss. We used Mann–Whitney (M–W) tests of ranked median threshold values from each hemisphere to compare the control and DCL groups, and we examined the distributions of motor threshold values between groups using Kolmogorov–Smirnov (K–S) two‐sample distribution tests. Statistical tests were performed in GraphPad Prism with alpha set to 0.05 for significance. Hypothesis testing was two‐tailed for differences from zero, except where noted.

## 3 Results

The main focus of this study was to characterize evoked motor response maps and determine if a unilateral transection of the somatosensory ascending pathway in the DC of the spinal cord affected the evoked movement representations and corticocortical connectivity in the deprived portions of contralateral somatosensory areas 3a, 3b, and 1, as well as the primary motor cortex (M1) after 1 year of postlesion recovery in squirrel monkeys. While somatosensory mapping is not a main focus here, it serves an essential purpose in defining rostrocaudal borders of area 3b, verifying the remaining neural responses to touch after long‐term recovery from sensory loss, and determining whether motor response functions in areas of the somatosensory cortex were correlated with the somatosensory responsiveness after sensory loss. We collected data from seven hemispheres of four squirrel monkeys: one normal monkey, and three monkeys with unilateral DCLs. The results are presented in four sections: (1) Evaluation of the extent of DCL; (2) General summary of somatosensory mappings; (3) Mapping of movements evoked by LT‐ICMS; (4) Corticocortical connections.

### 3.1 Evaluating the Extent of Dorsal Column Lesion

Characterizing the lesion extent is an important initial step to determine the impact of DCL on the hand region of the somatosensory cortex and motor response maps in the sensorimotor cortex.

**FIGURE 1:**
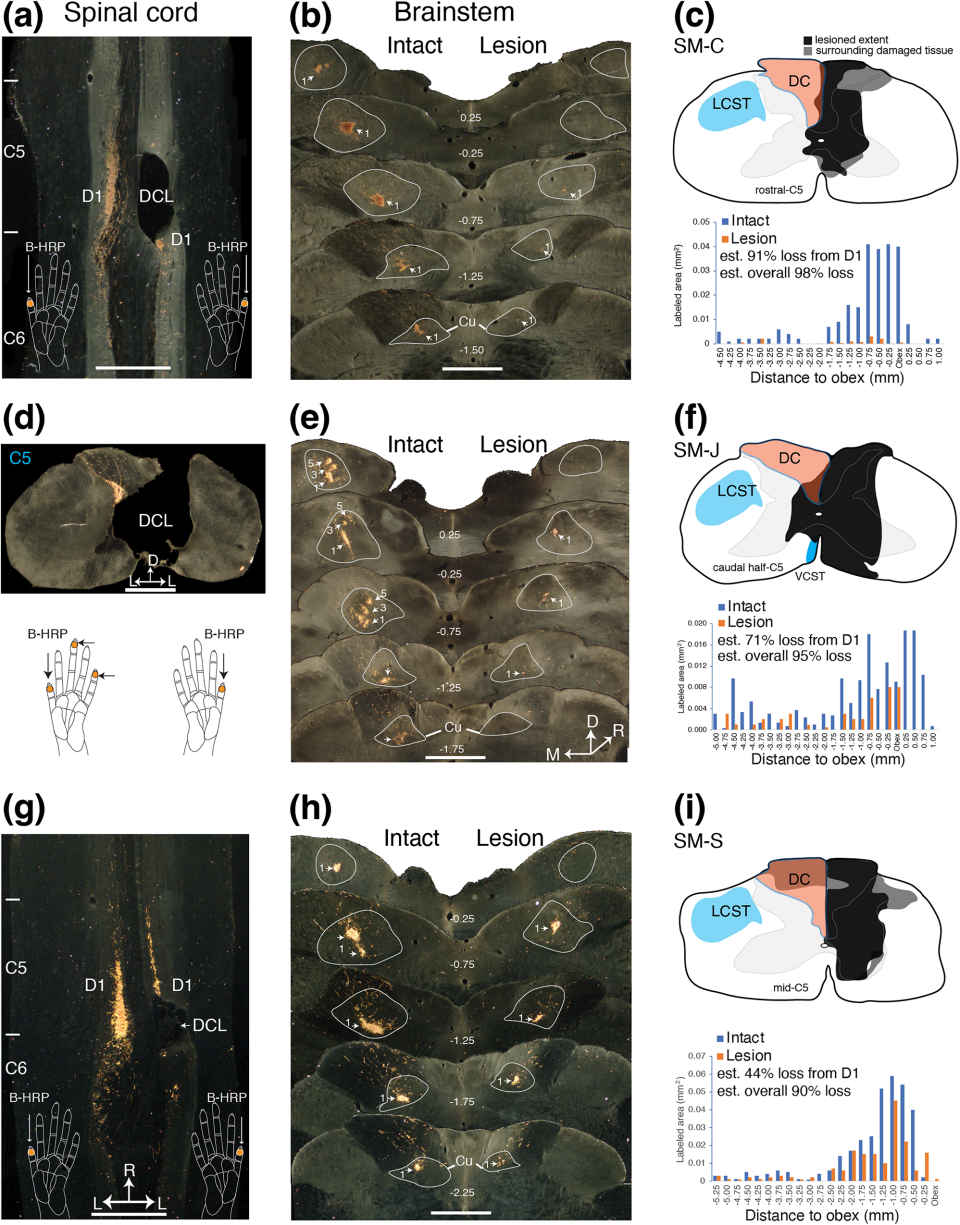
Evaluation of the extent of dorsal column lesion (DCL). The extent of DCL was evaluated by comparing the amounts of labeled peripheral nerve terminations in the cuneate nucleus (Cu) between the lesioned side (right) with that of the intact side (left). Axonal terminations were labeled by injections of cholera toxin subunit B conjugated to horseradish peroxidase (B‐HRP) into the distal phalange of digit 1 of both hands for monkeys SM‐C (a–c) and SM‐S (d–f), or digits 1, 3, and 5 of the left hand and digit 1 of the deafferented right hand for SM‐J (g–i). The locations of B‐HRP injection sites are indicated by dots on the schematic drawings of hands. (a, d) Photomicrograph of a tetramethylbenzidine (TMB)‐reacted horizontal section of the cervical spinal cord showing the location of the DCL and labeled axon terminals (pink patches) in the dorsal horns after tracer injections into the skin of distal digit 1 of both hands for monkeys SM‐C (a) and SM‐S (d). Cervical spinal cord locations are indicated on the left of photomicrographs (C5, C6). Note that lesions interrupted most of the peripheral afferents from digit 1 on the right side of the spinal cord. (g). Photomicrograph of a TMB‐reacted coronal section across the site of largest damage of the cervical spinal cord shows the location of the DCL for monkey SM‐J. (b, e, h) TMB‐reacted coronal sections through dorsal column (DC) nuclei of the brainstem of SM‐C (b), SM‐S (e), and SM‐J (h). The rostrocaudal section levels in relation to the distance from the obex are listed in mm. The cuneate nucleus is outlined in gray, and numbers 1 or 1, 3, and 5 mark foci of afferents labeled by B‐HRP injections into digits 1, 3, and 5. Foci of axon fibers in the Cu on the same side as the DCL in the spinal cord were reduced. (c, f, i) Transverse view of the spinal cord through cervical segment C5 indicating the reconstructed extent of the DCL in black. Dark gray shades adjoining the damaged tissue (black) likely represent scar tissue or cysts. DCL was reconstructed from the series of horizontally cut (a, d) or coronal (g) sections. Locations marked as lateral corticospinal tract (LCST) based on *Carpenter's Human Anatomy* 9th edition (1996). Bar graphs compare the areal sizes of BHRP‐labeled axon arbor foci in the Cu for the lesioned and intact sides. The *x*‐axis shows the distance (in mm) measured from the beginning of the obex, where negative values indicate distances caudal to the obex. The *y*‐axis value is the areal size (in mm^2^) of the combined foci of the B‐HRP‐label for each section through the Cu. C5–C6, fifth and sixth segments of the cervical spinal cord; Cu, cuneate nucleus of the lower brainstem; D1, digit 1; D, dorsal; DC, dorsal column; DCL, dorsal column lesion; L, lateral; LCST, lateral corticospinal tract; M, medial; R, rostral; VCST, ventral corticospinal tract. Scale bars, 1 mm for (a, b, d, e, g, h).

Reconstruction of damaged tissue in the spinal cord indicated that the lesions involved the cuneate pathway of the DCs, while also damaging a portion of the central gray matter at the lesion level for all three monkeys. Due to damage to the central gray, our lesions may have interrupted the C3–C4 intersegmental propriospinal network connections to the damaged segments. This pathway in primates contributes to postural control, head and neck movement coordination, reflex responses, sensorimotor integration, and fine motor control. The C3–C4 propriospinal neurons are involved in forelimb “target reaching” movements (Isa et al. 2007, review) and other forelimb and hand movements (Maier et al. 1998; Nakajima et al. 2000; see Lemon 2019; Lemon and Morecraft 2023 for review). The lateral corticospinal tract, one of the major motor pathways involved in manual dexterity in nonhuman primates, was mostly spared in all three monkeys (Figure 1).

#### 3.1.1 Monkey SM‐C

The unilateral DCL was on the right side of rostral cervical C5 of the spinal cord in this monkey, and only a few peripheral afferents from D1 were spared (Figure 1a). Reconstruction of the damaged site in the cervical spinal cord indicated that the lesion was complete or nearly so (schematic drawing in Figure 1c). Thus, most of peripheral afferents from digits 2 to 5 that entered the tract below the lesion were cut. A quantitative comparison of labeled axon terminal territories from two sides of cuneate nuclei was made after tracer injection into the matched locations on digits 1 of both hands. Results indicated that 91% of peripheral afferents from digit 1 were cut (bar graph in Figure 1c). Since the section at the C5 level was complete or near complete, we estimate that all 91% of ascending axons from digit 1 and nearly all axons from digits 2 to 5 and palm pads were interrupted as they entered below the cut. The proportion of peripheral ascending fibers from each digit to the upstream stations in the somatosensory system may or may not be equal. To our knowledge, there has been no study that directly measured the proportions of afferents from each digit in the cuneate nucleus of monkeys, as the cuneate nucleus is a long nucleus along the rostrocaudal axis in the brainstem, and it is heterogeneous in size and form. However, the proportion of digit representations in the hand region of somatosensory area 3b of squirrel monkeys has been reported, and results indicate that the proportion of the mean area of digits D1–D5 is 18%, 21%, 21%, 14%, 10%, respectively (Merzenich et al. 1987, see also Jain et al. 1998; Qi et al. 2021). Thus, combining anatomical measures from the current result (91% loss from D1) with the measures reported in the literature, the overall estimate of peripheral afferents lost from the hand for squirrel monkey SM‐C is 98% (9% × 18% = 1.6% of spared afferents). We use the overall sensory loss from the hand for this case (i.e., 98%) and other cases throughout the manuscript.

#### 3.1.2 Monkey SM‐J

Reconstruction of damaged sites in the cervical spinal cord for monkey SM‐J indicated that unilateral DCL was complete at this level (Figure 1d, and schematic drawing in Figure 1f), and the elongated lesion was through the lower half of C5, resulting in most of the peripheral afferents from digit 3 to 5 (below the lesion) being cut. A portion of peripheral afferents from digit 1 and maybe some from digit 2 entered above the lesion and thus were spared. Quantitative comparison for the labeled axon terminal territories from two sides of cuneate nuclei was less straightforward than in the other two monkeys. As part of another study, a B‐HRP tracer was injected into the tips of digits 1, 3, and 5 of the intact hand, and an equal amount of B‐HRP was injected into the matching site of digit 1 of the denervated hand. We estimated the percentage of ascending afferents lost in the cuneate nuclei by calculating the areal ratio of the labeled terminal field between the lesioned side and that of the intact side. The total labeled area from the lesioned side was divided by the mean value of the total labeled area from the three‐digit injections of the intact side, resulting in a 71% loss to the cuneate nucleus of the brainstem. Similarly, the overall estimate of peripheral afferents lost from the hand for squirrel monkey SM‐J was 95% (29% × 18% = 5% of spared afferents).

#### 3.1.3 Monkey SM‐S

A unilateral DCL was made on the right side of mid‐cervical C5 where some fibers from D1 were spared (Figure 1g). The reconstruction of the damaged site indicated that the lesion was nearly complete (schematic drawing in Figure 1i). A quantitative comparison of labeled axon terminal territories from two sides (lesioned and nonlesioned) of cuneate nuclei indicated that 44% peripheral ascending afferents from digit 1 to cuneate nucleus were lost (bar graph in Figure 1f). Compared to the similar nearly complete DCL in monkey SM‐C, the lesion at the slightly lower level in cervical C5 of SM‐S resulted in more afferents from D1 (56%) and likely some axons from digit 2 projecting to the cuneate nucleus by entering above the lesion than those of SM‐C. Using the same estimation equation as for monkey SM‐C, the overall estimate of peripheral afferents lost from the hand for squirrel monkey SM‐S was 90% (56% × 18% = 10% of spared afferents).

The current study using anatomical measurements to estimate the extent of DCL by tracer injections into one digit for both hands is less informative compared to our early studies (e.g., Qi et al. 2011a, 2021). However, including the electrophysiological recordings of neuronal receptive fields and somatotopy in area 3b is a complete approach to determining the effectiveness of sensory loss because of the precise somatotopic features in areas 3b and 1 of primates (Merzenich et al. 1978; Sur et al. 1982). In the following section, we further describe mapping results case‐by‐case, starting from the most severe DCL case after summarizing the normal somatosensory map properties.

### 3.2 Somatosensory and Motor Mapping of Sensorimotor Areas M1, 3a, 3b, and 1

#### 3.2.1 Somatosensory and Motor Mapping in Normal Sensorimotor Cortex

To distinguish sensorimotor cortex areas and the topography of receptive fields or evoked motor responses, we performed somatosensory mapping, followed by motor mapping. Under general anesthesia, neuronal responses and receptive field properties to tactile stimulation were characterized using multiunit microelectrode recording. The hand regions of somatosensory areas 3a, 3b, and 1 of the left hemispheres in one control monkey and the deprived side of three lesioned monkeys were mapped after 11–13 months of recovery from unilateral DCL.

Motor mapping in sensorimotor cortex was carried out with LT‐ICMS applied to the forelimb regions of sensorimotor areas M1, 3a, 3b, and 1. Movements were evoked from the body contralateral to sites of LT‐ICMS in all cases. No ipsilateral or bilateral movements were observed. Note that the trunk and hindlimb territories were not a major focus of this study, and therefore, were not explored.

**FIGURE 2:**
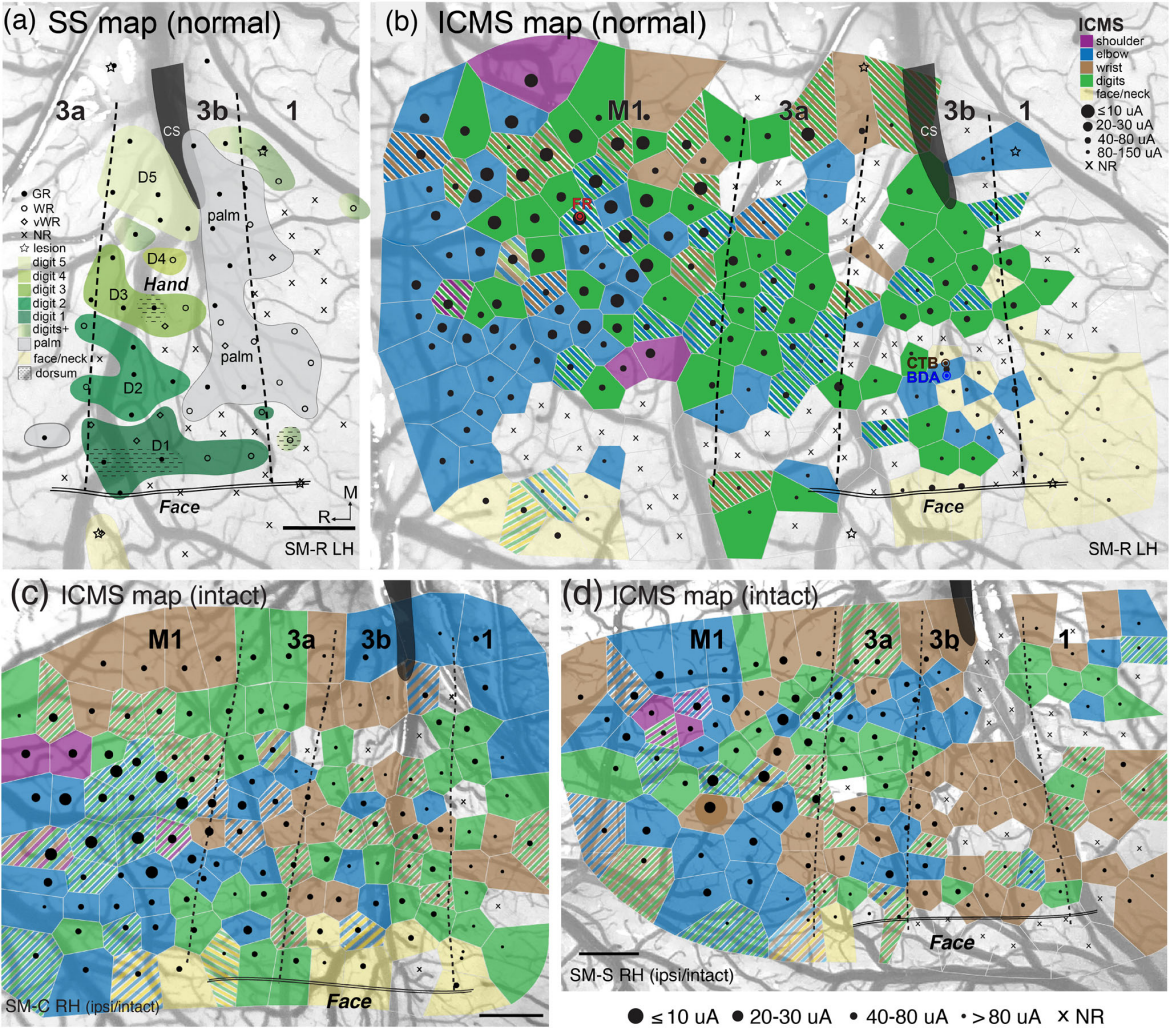
Electrophysiological maps of sensorimotor cortex. (a). Surface view of left sensorimotor cortex showing somatosensory map (SS) and long‐train (500 ms) intracortical microstimulation (LT‐ICMS) map for control squirrel monkey SM‐R. In the hand region of area 3b, small receptive fields produced by light touch reveal the typical somatotopic order of the hand representation with the digits 1–5 (dark to light green) arranged in a lateral to medial sequence in the rostral part of area 3b, and palm representation in the caudal part of 3b. Solid dots mark numbered electrode penetrations with good responses; black open circles mark those with weak responses; black diamonds mark those with very weak responses to hard taps; x marks penetrations with no responses. Dotted patterns mark representations of hairy skin of the hand. (b). Upon obtaining the somatosensory map, a biphasic LT‐ICMS map was made throughout the hand and forelimb regions of areas 3a, 3b, 1, 2, and M1. Results depicted with Voronoi tile color correspond to types of threshold‐elicited movements of joints. Combined joint movements evoked are indicated by combinations of colored stripes. Evoked digit, wrist, elbow, and shoulder movements are commonly seen in the forelimb region of M1, and they are also present in the hand regions of somatosensory areas 3b and 3a. The excitability to evoke movements at each site is indicated by the size of the black dots based on threshold currents (10–150 µA). Sites with thresholds beyond 150 µA are considered nonexcitable and marked with “x.” Thresholds to evoke visible movements are generally lower in M1 (high excitability, larger dots) than those in the somatosensory cortex (small dots). (c) and (d). LT‐ICMS maps from the right hemisphere of two intact sides of DCL monkeys (SM‐C and SM‐S, respectively). Dashed lines represent areal borders, double lines indicate hand–face border of area 3b, and stars mark electrolytic lesion sites for aligning electrophysiological boundaries with histologically processed tissue. 3a, 3b, 1, 2, somatosensory areas 3a, 3b, 1, and 2; BDA, biotinylated dextran amine; CTB, cholera toxin subunit B; cs, central sulcus; D1–D5, digit 1–5; DCL, dorsal column lesion; FR, Fluororuby; GR, good response; ICMS, intracortical microstimulation; LCST, lateral corticospinal tract; LH, left hemisphere; LT, long‐train; M, medial; M1, primary motor cortex; NR, no response; SS, somatosensory cortex; R, rostral; vWR, very weak response; WR, weak response. Scale bars, 1 mm.

Similar to previous findings in normal squirrel monkeys (Sur et al. 1982) and monkeys with DCL (Qi et al. 2011a, 2016, 2021), somatotopic maps of digits and palm with strong responses to tactile stimulation were found in control hemispheres, where digits 1–5 were represented in a lateromedial progression in area 3b, with distal digits located rostrally and proximal digits and palm pads located caudally. The left and right hemispheres of monkey SM‐R were mapped as normal control samples, with the left hemisphere mapped more densely. Figure 2a depicts a somatosensory map in the left hemisphere of monkey SM‐R, in which 36 out of 45 (80%) mapping sites in the hand region of area 3b responded to light touch on the digits and hand sites tested.

Figure 2b shows the motor map of the left hemisphere of normal monkey SM‐R. The threshold‐elicited movements of digits, wrist, elbow, and occasionally shoulder were found throughout the forelimb region of sensorimotor areas M1, 3a, 3b, and 1. Digit movements, either single or multiple digits, were widely evoked in M1. Results from the right hemisphere of this monkey were similar to those shown in Figure 2b. The topographic organizations of M1 in squirrel monkeys were consistent with previous findings.

Digit movements were also evoked from areas 3a, 3b, and occasionally in area 1 when current up to 150 µA was applied. Like those in M1, the evoked digit movements in somatosensory cortex were most often intermingled with the evoked movements of the wrist, elbow, and arm. Sites that responded to LT‐ICMS with combined movements of digits, wrist, forearm, or shoulder are depicted by various stripes in Figure 2b. Overall, digit movements produced by LT‐ICMS were distributed in mosaic and complex fashions in squirrel monkeys.

Figure 2c,d illustrates motor maps made from the intact side of the cortex (ipsilateral to DCL) for monkeys SM‐C and SM‐S. These two maps of the right hemispheres were flipped along the horizontal axis for easier comparison with that of normal monkey (Figure 2b). Data from the intact side of monkey SM‐J were not available. Overall, the excitability and topographies of evoked movements throughout sensorimotor areas were similar between the intact cortices of lesioned monkeys (Figure 2c,d) and the normal hemispheres (Figure 2b; the motor map from the right hemisphere of this monkey is not shown).

To increase the sample of data from M1, we added a subset of available data using ST‐ICMS in squirrel monkeys (Wu and Kaas 1999). Three key reasons support this addition. (1) The studies were from the same laboratory, same animal species, and under the same anesthetized experimental conditions. (2) ST‐ICMS is an initial step of LT‐ICMS, and the ICMS durations (50 ms vs. 500 ms) do not affect the evoked muscle activation pattern (e.g., Capaday 2022). (3) The motor maps of M1 in intact hemispheres using ST‐ICMS and LT‐ICMS showed 100% of tested sites were responsive to ICMS, and thus, showed no difference in responsiveness. ICMS motor maps of somatosensory cortex areas were not available from prior publications to add to our dataset. No significant difference was detected between these two studies for the ratio of evoked digit‐related movement versus the rest of forelimb movements (i.e., wrist, elbow, arm, and shoulder, and any of their combinations) in the combined M1 control groups (Friedman = 4.87, *p* = 0.5606). Thus, we added two aspects of the data from the early study, (1) responsiveness, and (2) the ratio of distal versus proximal forelimb movements obtained from M1 of a normal control squirrel monkey and two intact hemispheres of squirrel monkeys after arm amputation (Wu and Kaas 1999).

#### 3.2.2 Somatosensory and Motor Mapping in the Deprived Sensorimotor Cortex

**FIGURE 3:**
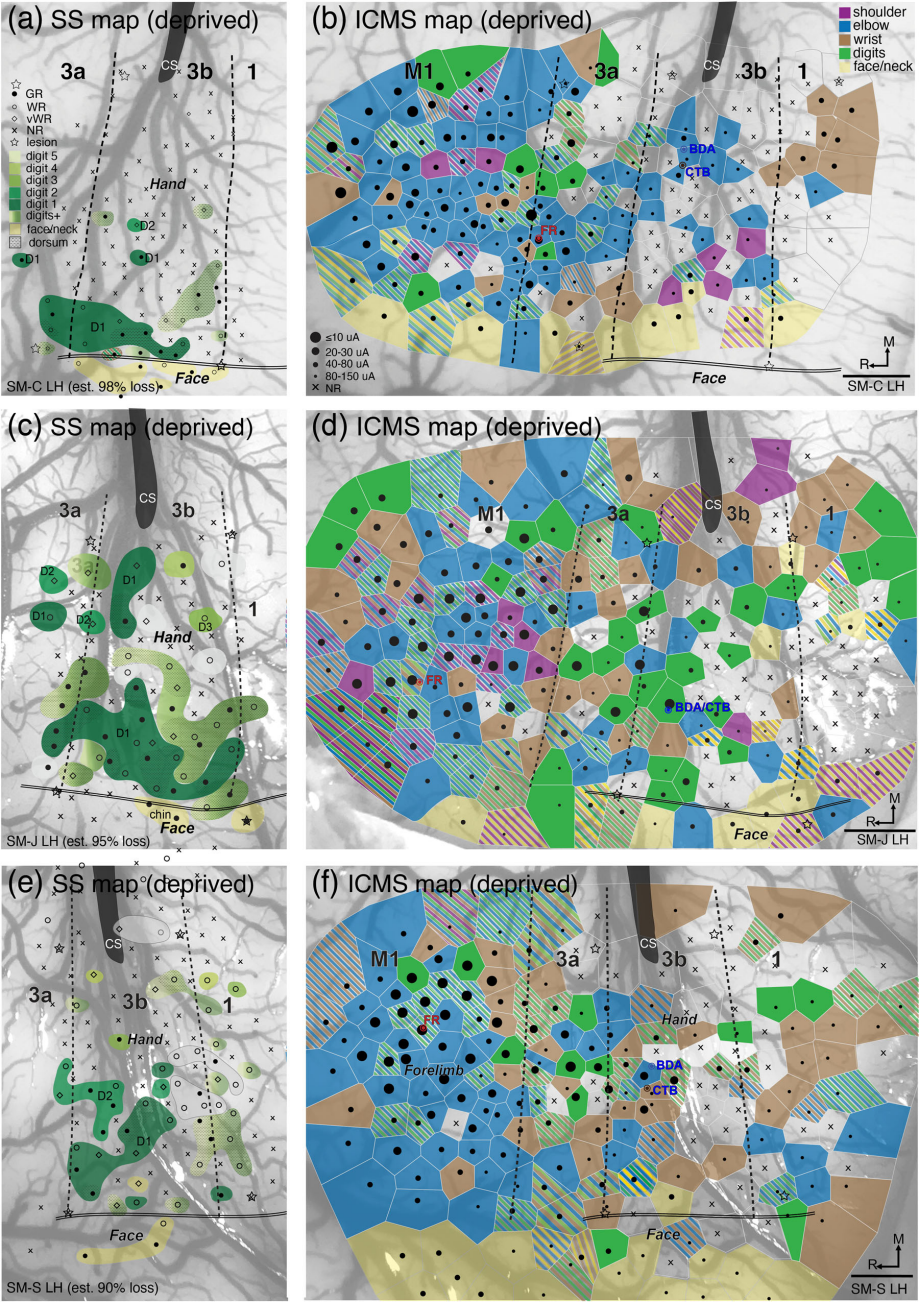
Electrophysiological maps of deafferented sensorimotor cortex contralateral to DCL. (a) Somatosensory map of neuronal receptive fields using microelectrode recordings in area 3b of monkey SM‐C 11 months after extensive DCL (est. 98% loss) shows neurons at a majority of mapping sites were not responsive to touch on digits or palm, except for a few sites representing spared D1 and D2. (b) Microstimulation map throughout areas 3a, 3b, 1, 2, and M1 of monkey SM‐C depicted with Voronoi tile colors corresponding to types of threshold‐elicited movements of joints. Note that after long‐term recovery from extensive DCL, more sites in areas 3b and 1 responded to LT‐ICMS with movements of proximal parts of forelimb, and evoked digit movements were mostly eliminated, corresponding with sensory loss from hand. While M1 remained highly excitable, the number of stimulation sites with evoked digit movements was reduced. (c). Somatosensory map of neuronal receptive fields in area 3b of monkey SM‐J 13 months after extensive DCL (est. 95% loss) shows that the partially spared D1 and D2 representations are highly responsive in area 3b. Neuronal responses are somewhat somatotopically organized, except that in the medial portion of the hand region, a few abnormal sites where neurons responded to touch on D1 are present. (d) Microstimulation map throughout areas 3a, 3b, 1, 2, and M1 of monkey SM‐J. (e) Somatosensory map of neuronal receptive fields in area 3b of monkey SM‐S 12 months after extensive DCL (90% loss) shows a majority of mapping sites did not respond to touch on digits or palm, except for a few penetration sites representing spared D1 and D2. (f) Microstimulation map throughout areas 3a, 3b, 1, 2, and M1 of monkey SM‐S shows areas 3b, 1, and 2 were responsive to electrical stimulation, and areas 3a and M1 were highly responsive. Evoked digit movements were somewhat reduced throughout the sensorimotor areas. Dashed lines represent borders of electrophysiologically defined cortical areas, double lines indicate the hand–face border of area 3b, and stars mark electrolytic lesion sites for relating electrophysiological results to histologically processed tissue. For abbreviations and conventions, see Figure 2. Scale bars, 1 mm for (b, d, f).

With severe sensory loss (est. 98% loss) from the right hand in monkey SM‐C, only a few recording sites had neurons that responded to touch on glabrous or dorsum digit 1, and occasionally on digit 2. Most neurons in the hand region were not responsive to tactile stimulation, as only 20 out of 69 (29%) recording sites in the hand region of area 3b responded to touch on the digits and hand (Figure 3a). Digit‐related movements appeared to be rarely present in affected areas 3b and 1, and they appeared to be reduced in M1 and 3a by qualitative assessment (Figure 3b).

In the second lesioned monkey SM‐J, who also had extensive DCL (est. 95% loss), neurons in the deprived hand region of somatosensory area 3b were highly responsive to tactile stimulation on digit 1 (Figure 3c) and some weak responses were also present from touch on digits 2 and 3. Abnormal somatotopy was shown by responses to touch on digit 1 that extended medially into the digits 3–5 territories, indicating the potential of axon sprouting in this afferent‐deprived area. Similarly, dorsum hairy skin was overrepresented. Overall responsiveness to touch in the hand region of area 3b in SM‐J was 66% (39/59). The evoked movement representations in the deprived somatosensory areas of this monkey (Figure 3d) were qualitatively similar to that of the control group. Elicited digit movements were found in multiple sites in areas M1, 3a, 3b, and 1, and sites for movement of proximal joints such as wrist, elbow, and shoulder surrounded them. One unusual pattern for this monkey was that movements from the shoulder alone or in combination with distal forelimb movements were found in the center of the distal forelimb region in M1.

In the third lesioned monkey SM‐S with extensive DCL (est. 90% loss from hand), 34 out of 67 (51%) mapping sites where neurons in the deprived hand region of area 3b responded to touch on digits and hand. Some sites where neurons responded to touch on digits 1 and 2, and more neurons responded to touch/stroke hairs on the dorsum skin (Figure 3e). In contrast to the results from somatosensory mapping, evoked digit‐only movements appeared to be rarely found in area 3b. Instead, digit movements were most often concurrent with the elbow and wrist in area 3b (stripes, Figure 3f). Evoked digit‐only movements (green) were present in areas 3a, 1, and M1.

We examined whether the evoked motor responsiveness of area 3b was related to the effect of the somatosensory deprivation due to the lesion, or if the evoked motor responsiveness of area 3b was related to the motor responsiveness of M1. We examined the relationship of responsiveness to tactile stimulation and to LT‐ICMS in area 3b for the most extensively mapped hemisphere in our normal and DCL cases (*n* = 4 pairs). The motor responsiveness was highly correlated with the somatosensory responsiveness in area 3b, with ranked values monotonically related (Spearman's rho = 1.0, *p* = 0.0417). There was no relationship between the motor responsiveness of M1 and that of area 3b (Spearman's rho = −0.40, *p* = 0.75) because the responsiveness of M1 was not significantly affected by DCL, while motor responsiveness of area 3b varied.

In summary, after long‐term recovery from a unilateral sensory loss from the hand, the proportion of sites evoking digit movements (Figure 3b,d) appeared to be reduced in areas 3a, 3b, and 1, but the trend did not reach statistical significance for our sample. The evoked movement patterns in M1 of the normal monkey and the intact sides of the lesioned monkey were similar (Figure 2), statistically indistinguishable, and consistent with the results of other studies. Overall, the responsiveness of somatosensory maps in area 3b of a normal monkey and three monkeys after DCL was correlated with the motor responsiveness in 3b, suggesting that somatosensory reorganization in area 3b likely contributed to the responsiveness of motor response maps in somatosensory areas. Thus, neurons with nearly normal responses to tactile stimulation appeared to be required for evoked motor responses from area 3b.

#### 3.2.3 Proportion of Elicited Distal Versus Proximal Forelimb Movements

To determine whether evoked digit movements were specifically reduced due to sensory loss from the digits and hand, we calculated the proportions of elicited movements involving digits (D&D+) and forelimb movements that were proximal to digits (A+), which included evoked movements of wrist, elbow, shoulder, and their combinations. The percentage of body movements was calculated for each cortical area (M1, 3a, 3b, and 1).

**FIGURE 4:**
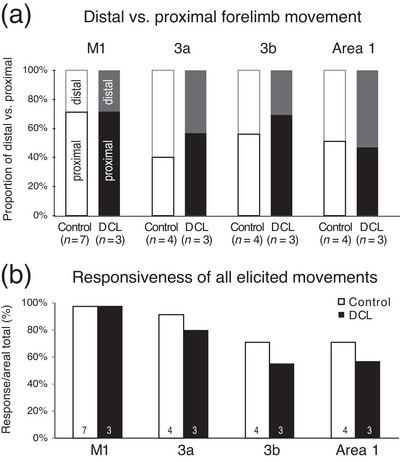
Comparing control and DCL groups in the proportion of evoked body movements and responsiveness to LT‐ICMS. (a) Proportions of movements involving distal digits or proximal forelimb movements are expressed in percentage (%) on the *y*‐axis, shown in stacked bar chart sets by cortical areas and lesion group. Numbers in parentheses in each group are the number of hemispheres studied. The proportion of evoked distal digit movements to forelimb movements tended to be lower in area 3a and area 3b for the DCL group, but a significant difference was not detected. (b) Comparison of the responsiveness of all elicited movements by areas for control and DCL groups shows a trend for lower responsiveness between control and DCL groups in 3a and 3b, but this was not significantly different when the proportions were analyzed using one value per hemisphere as the subject unit of measure. The number of hemispheres studied is labeled for each column. For abbreviations and conventions, see Figures 1 and 2.

Figure 4a depicts the ratio of the evoked movement involving digits (D&D+) and evoked forelimb movements that involved wrist, elbow, and arm/shoulder (A). When we tested the ratio of evoked digit‐related movement sites to the rest of the evoked forelimb movement sites as a value for each hemisphere, we again could not detect differences between hemispheres for each cortical area (M1 K–W = 0.007148, *p* > 0.9999; for all other areas K–W = 0.0000, *p* > 0.9999). Thus, we did not detect an effect of DCL when using the hemisphere as the subject unit of measure for motor responsiveness and for the ratio of digit movement sites to other evoked movement sites. Although we did not detect differences between hemispheres, regardless of DCL, we plotted the mean ratios for the normal and intact hemispheres as a control group to compare visually with the DCL group. The proportion of proximal movements appeared to be greater than that of distal digit movements in areas 3a and 3b for the DCL group (black vs. gray filled bars) compared to the control group (black vs. gray outlines), without reaching statistical significance (Figure 4a). Subsequent figures and analyses use the same conventions when group results are depicted.

#### 3.2.4 Excitability to Electrical Stimulation Across Sensorimotor Cortex

We analyzed excitability to LT‐ICMS in two ways: (1) responsiveness, which was simply calculated by the total number of responsive sites to the maximum current intensity (150 µA) or less, divided by the total stimulation sites within each sensorimotor area (M1, 3a, 3b, and 1); (2) the threshold amplitudes determined by the minimum current intensity used to evoke body movements.

##### 3.2.4.1 Responsiveness to LT‐ICMS

In the present study, a total of 1188 sites were stimulated with LT‐ICMS from seven hemispheres of four monkeys (Table 3). Among those, 944 sites were responsive, and 244 sites were nonresponsive. In nondeprived control cortices (normal and intact), the responsiveness gradually decreased across sensorimotor areas from M1 to 3a, 3b, and 1 with the most responsive area being M1 and the least responsive areas being 3b and 1 (Figure 4b, gray bars). This downward trend in responsiveness across sensorimotor areas was also found in the lesioned group (Figure 4b, black bars). In M1, to increase sample sizes in the control group, as mentioned earlier, we added a subset of data on responsiveness from previously published data using ST‐ICMS in squirrel monkeys (Wu and Kaas 1999). We examined the data using hemisphere as the unit of measure, with 10 hemispheres for M1 and seven hemispheres for all other areas. When testing the proportion of sites responsive to LT‐ICMS in this way, we did not detect significant differences between normal hemispheres, intact hemispheres of lesioned monkeys, and deprived hemispheres for areas M1, 3a, 3b, and 1 (K–W = 0.0000, *p* > 0.9999 for all areas), despite the trend for less responsiveness in somatosensory areas after DCL (Figure 4b).

**TABLE 3:** Number of stimulation sites per case.

Case	M1 (response/NR)	3a (response/NR)	3b (response/NR)	Area 1 (response/NR)	Total (ICMS sites)
SM‐R LH (normal)	79/13	28/7	37/23	16/8	211
SM‐R RH (normal)	26/0	26/2	19/8	16/9	106
SM‐C LH (deprived)	74/2	35/14	26/34	9/9	203
SM‐C RH (intact)	47/0	25/1	39/4	16/2	134
SM‐J LH (deprived)	76/2	28/4	35/22	19/16	202
SM‐S LH (deprived)	63/1	25/6	26/17	26/13	177
SM‐S RH (intact)	48/0	28/1	30/17	22/9	155
98‐62 LH (ST‐ICMS, normal)	74/0	x	x	x	74
98‐61 RH (ST‐ICMS intact)	89/0	x	x	x	89
98‐64 LH (ST‐ICMS intact)	87/0	x	x	x	87

Abbreviation: NR, no response. Other abbreviations are consistent with those in Figure 7.

##### 3.2.4.2 Comparing the Mean Threshold Value Across the Sensorimotor Cortex

The mean threshold intensity for evoking body movement is another crucial factor in comparing the excitability between sensorimotor areas. Threshold current was defined as the current level where a just‐detectable movement occurred on at least half of the trials applied to a stimulating site. We typically started with a current level above the expected threshold for a detectable movement and then reduced it until a movement was no longer detected. On the occasions when no movement was initially detected, current levels were gradually raised until a movement was observed or 150 µA was reached.

**FIGURE 5:**
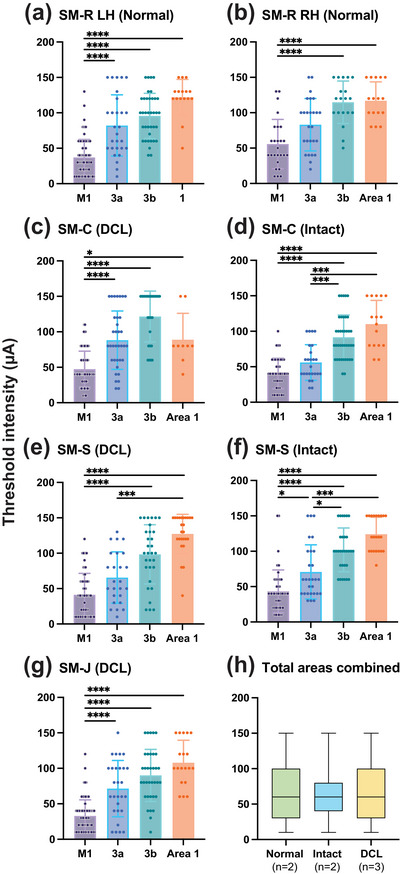
Comparisons of the mean threshold currents of all evoked body part movements across four cortical areas of each studied case (a–g). Mean threshold and *SD* are indicated on the *y*‐axis and sensorimotor areas on the *x*‐axis. Symbols on top of columns show statistically significant differences in mean thresholds (K–W test, *p < 0.05; ***p < 0.001; ****p < 0.0001). (h) Box plots depict the distributions of threshold current intensity used to evoke movement from seven hemispheres combined into normal, intact, and DCL groups for all cortical areas. Horizontal lines indicate the medians and boxes indicate the interquartile range. Numbers in parentheses in each group are the number of hemispheres. LH, left hemisphere; RH, right hemisphere. For other conventions, see Figure 2.

Figure 5 shows the threshold current for eliciting body movements from the forelimb region of the seven hemispheres. Among these, four motor maps were obtained from control hemispheres (Figure 5a,b,d,f), and three motor maps were obtained from sensory‐deprived hemispheres (Figure 5c,e,g). The forelimb regions of M1 were highly responsive, as reflected by the lowest mean threshold current needed to evoke body movements. The mean threshold current required to evoke movement tended to gradually increase across somatosensory areas 3a, 3b, and 1 in six out of seven hemispheres. The exception was the deprived hemisphere of monkey SM‐C, in which the mean threshold of area 1 appeared to be lower than that of area 3b and was similar to that of area 3a (Figure 5c). Due to the low number of sites sampled from area 1, we eliminated area 1 from the repeated measures test of motor thresholds with the seven subjects (hemispheres) and the three areas (M1, 3a, 3b) as the groups. The test with Dunn's adjustment for multiple comparisons detected that thresholds in M1 were lower than in area 3b, regardless of hemisphere or DCL condition (Friedman test = 14.0; *p* = 0.0005), while the thresholds for area 3a appeared to be between M1 and 3b and not statistically different (*p* = 0.1841 for both pairwise comparisons). Thus, the progressive increase of mean thresholds from M1 to 3b was consistent in the seven studied hemispheres, with and without DCL.

##### 3.2.4.3 Comparing the Mean Threshold Value Between Lesioned and Nonlesioned Groups

To determine whether DCL affected the elicitability overall, as reflected by the threshold current in the forelimb region of the sensorimotor cortex, group comparisons were performed without distinguishing between cortical areas. Figure 5h shows that the distributions of threshold current intensities for evoked movements among normal, intact, and DCL groups were similar. Statistical comparisons of threshold current intensities with the K–W test indicated that there was no significant difference among the three groups (K–W = 1.548, *p* = 0.461).

**FIGURE 6:**
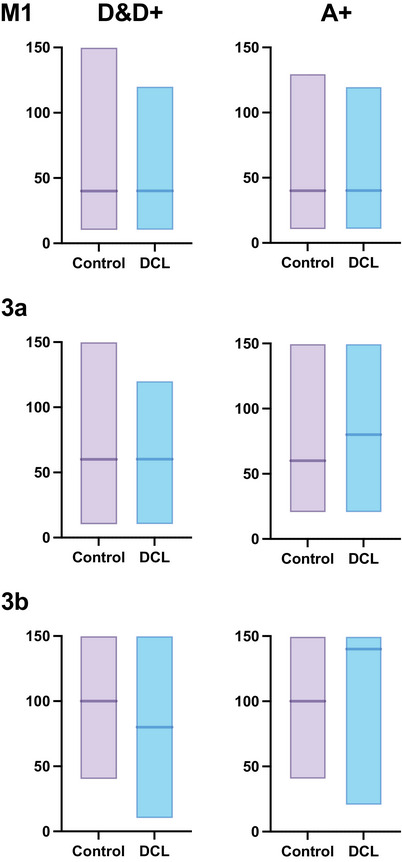
Current thresholds to evoke digit group (D&D+) and proximal forelimb group (A+) movements for each cortical area, comparing the control group (control + intact) and the DCL group. Horizontal lines indicate the medians. The numbers of hemispheres for the D&D+ and A+ groups are: M1control n=7, DCL n=3; 3a control n=4, DCL n=3; 3b control n=4, DCL n=3. Abbreviations, D, digit(s), D+, concurrent movements of digit and forelimb joints; A+, evoked forelimb movement proximal to digit(s); F/N, face and/or neck. For other conventions, see Figure 2.

To further determine whether the DCL altered the mean thresholds for evoking movements of a specific body part, we compared the differences in the mean thresholds between the control group and that of the lesion group in two categorized movement groups: evoked movements involving digits (D&D+), and evoked movements that were proximal to the digit (A+, i.e., wrist, elbow, shoulder). To do this, we first tested if data from two hemispheres of the normal monkey were different from that of the intact (ipsilateral) side of the cortex of two lesioned monkeys (data from the lesioned monkey SM‐J were not available). Figure 6 depicts the mean thresholds required for each movement category across sensorimotor areas. Note that data from area 1 were not included in the calculation due to the small sample size. We did not detect any significant differences using the M–W test to compare the ranked mean threshold values from control and DCL hemispheres for each cortical area by body part group. Thus, there was no significant difference in the thresholds of evoked movements for digits (D&D+ M–W = 4.0, *p* = 0.6286) or arm (A+ M–W = 4.0, *p* = 0.6286) in M1; area 3a (D&D+ M–W = 2.0, *p* = 0.2286; A+ M–W = 4.0, *p* = 0.6286); and area 3b (D&D+ M–W = 3.0, *p* = 0.40; A+ M–W = 3.0, *p* = 0.40). However, when examining the distribution of threshold values, the K–S test between the control group and the DCL group indicated that the distribution of thresholds for evoking arm movements in the lesion group were significantly higher than that of the control group (A+ K–S test = 0.4166, *p* = 0.0003) for area 3b.

In summary, the hand regions of sensorimotor areas M1, 3a, 3b, and 1 were responsive to LT‐ICMS after 11–13 months of recovery from sensory loss in squirrel monkeys, with some noted differences from normal.

### 3.3 Corticocortical Connections

It was also important to determine if cortical neurons of the hand representations changed their connections in response to sensory loss and long‐standing deprivation. The anatomical results are described in some detail here, but the main point is that the connections are similar to those in the cortex with normal somatosensory inputs.

Injecting different tracers in the forelimb territories of M1 and area 3b in the same monkey provided an opportunity for direct comparisons of the cortical connections of these two hand regions. To this end, we injected neuroanatomical tracer BDA along with CTB into the electrophysiologically defined forelimb regions of area 3b (referred to together as BDA and CTB) and FR into forelimb regions of M1. For details of the injection sites, see Table 2. Labeled neurons were plotted in sections of flattened cortex cut parallel to the cortical surface, which allowed us to superimpose the anatomical connection results and physiological maps in the same plane. Although the tracers BDA‐, CTB‐, and FR‐labeled neurons that projected to and from the injection sites, the retrogradely labeled cell bodies were more readily visualized than anterogradely labeled terminals. Thus, we focused on the distribution patterns of retrogradely labeled neurons. The evoked movement regions of M1 and 3b were injected with different tracers in three of the four monkeys (SM‐R, SM‐J, SM‐S). For DCL monkey SM‐C, the connection patterns of evoked elbow movement regions of area 3a and area 3b were injected with different tracers.

#### 3.3.1 Corticocortical Connections After Tracer Injections in Sensorimotor Cortex

**FIGURE 7:**
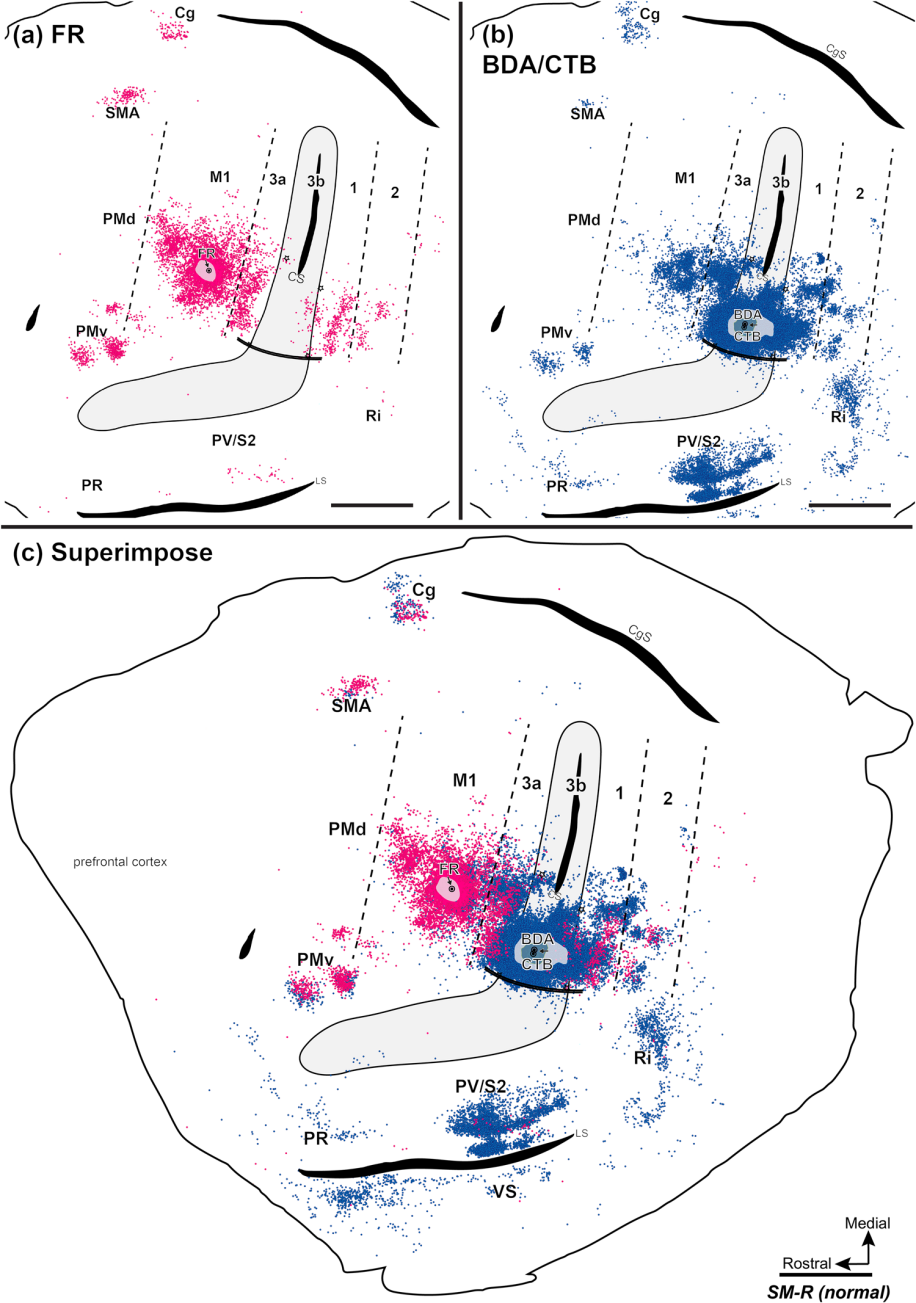
Distribution of labeled neurons from FR injection into the M1 digit and elbow movement zone (a, red) and BDA and CTB injections into the area 3b elbow movement zone (b, blue) in a normal monkey SM‐R. Each dot represents labeled neurons. Injection cores are indicated in shades of red and blue in the center of injection sites, dense uptake zones are represented in light shading where individual neurons cannot be counted. (c) The illustration is a composite of multiple flattened sections that were aligned based on anatomic landmarks such as the central sulcus dimple, tissue artifacts, and blood vessels. Dashed lines represent areal borders. The designation of rostral and caudal borders of area 3b and the hand/face border (solid lines) were determined from electrolytic lesions (stars) made at the end of electrophysiological mapping and VGLUT2 immunoreaction in adjacent cortical sections. Neurons labeled from 3b injections are extensively distributed in somatosensory areas 3b, 1, 3a, S2, and PV; less dense clusters of neurons are labeled in the expected locations of M1, PMd, PMv, PR, Ri, VS. Neurons labeled from M1 injection are extensively distributed in M1, with less dense clusters of labeled neurons distributed in 3a, 1, PMd, PMV, SMA, and Cg; largely avoiding area 3b. Populations of neurons projecting to 3b (blue) and projecting to M1 (red) largely overlapped in area 3a, 1, 2, PMd, PMv, and Cg. 3a, 3b, 1, 2, somatosensory areas 3a, 3b, 1, and 2; BDA, biotinylated dextran amine; Cg, cingulate gyrus region; CgS, cingulate sulcus; cs, central sulcus; CTB, cholera toxin subunit B; FR, Fluororuby; M1, primary motor cortex; PMd, dorsal premotor cortex, PMv, ventral premotor cortex; PR, parietal rostral cortex; PV, parietal ventral somatosensory area; Ri, retroinsular cortex; S2, secondary somatosensory area; SMA, supplementary motor cortex; VGLUT2; vesicular glutamate transporter type 2; VS, ventral somatosensory area. Scale bars, 5 mm.

In the control monkey SM‐R, BDA and CTB were placed in a site in the hand region of area 3b, where LT‐ICMS elicited elbow movement, and neurons at this site responded to touch on the palm (Figure 2a). FR was injected into the forelimb region of M1, where LT‐ICMS evoked concurrent movements of digits and elbow. BDA‐ and CTB‐labeled neurons projecting to the area 3b hand region were found in expected cortical locations related to sensorimotor function (Figure 7a). In addition to dense labeling within area 3b, dense distributions of BDA‐ and CTB‐labeled neurons were also present in area 3a, the caudal portion of M1, and in secondary somatosensory area (S2) and parietal ventral area (PV) along the upper bank of the lateral sulcus. Scatterings of BDA‐ and CTB‐labeled neurons projecting to area 3b were observed in area 2, ventral premotor area (PMv), supplementary motor area (SMA), cingulate gyrus (Cg) region, parietal rostral area (PR), retroinsular cortex (Ri). A few labeled neurons were in the ventral somatosensory area (VS), and very few were in the dorsal premotor area (PMd).

For projections to M1 after FR injection in the M1 digit and arm movement region, in contrast, only a few FR‐labeled neurons were found in somatosensory area 3b, with slightly more in S2 (Figure 7b). However, dense clusters of FR‐labeled neurons were spread throughout most of the forelimb region of M1 and nearby 3a. Less dense but focused groups of labeled neurons appeared in the expected forelimb part motor areas PMv, SMA, Cg, and somatosensory areas 1 and 2. These results align with previous results from monkeys with intact somatosensory inputs (Jones et al. 1978; Krubitzer and Kaas 1990; Darian‐Smith et al. 1993; Stepniewska et al. 1993; Wu and Kaas 2003; Gharbawie et al. 2011).

Beyond the injection cores and dense uptake zones, the two populations of labeled neurons from two tracer injections overlapped in several regions of cortex (Figure 7c). Clusters of FR‐labeled neurons projecting to M1 and BDA‐ and CTB‐labeled neurons projecting to 3b co‐localized in expected motor areas PMv, SMA, and Cg region, and somatosensory areas 1, 2, S2, and PV. Scattered overlaps of clusters of neurons projecting to the injection sites in area 3b and M1 were found in PR and Ri.

**FIGURE 8:**
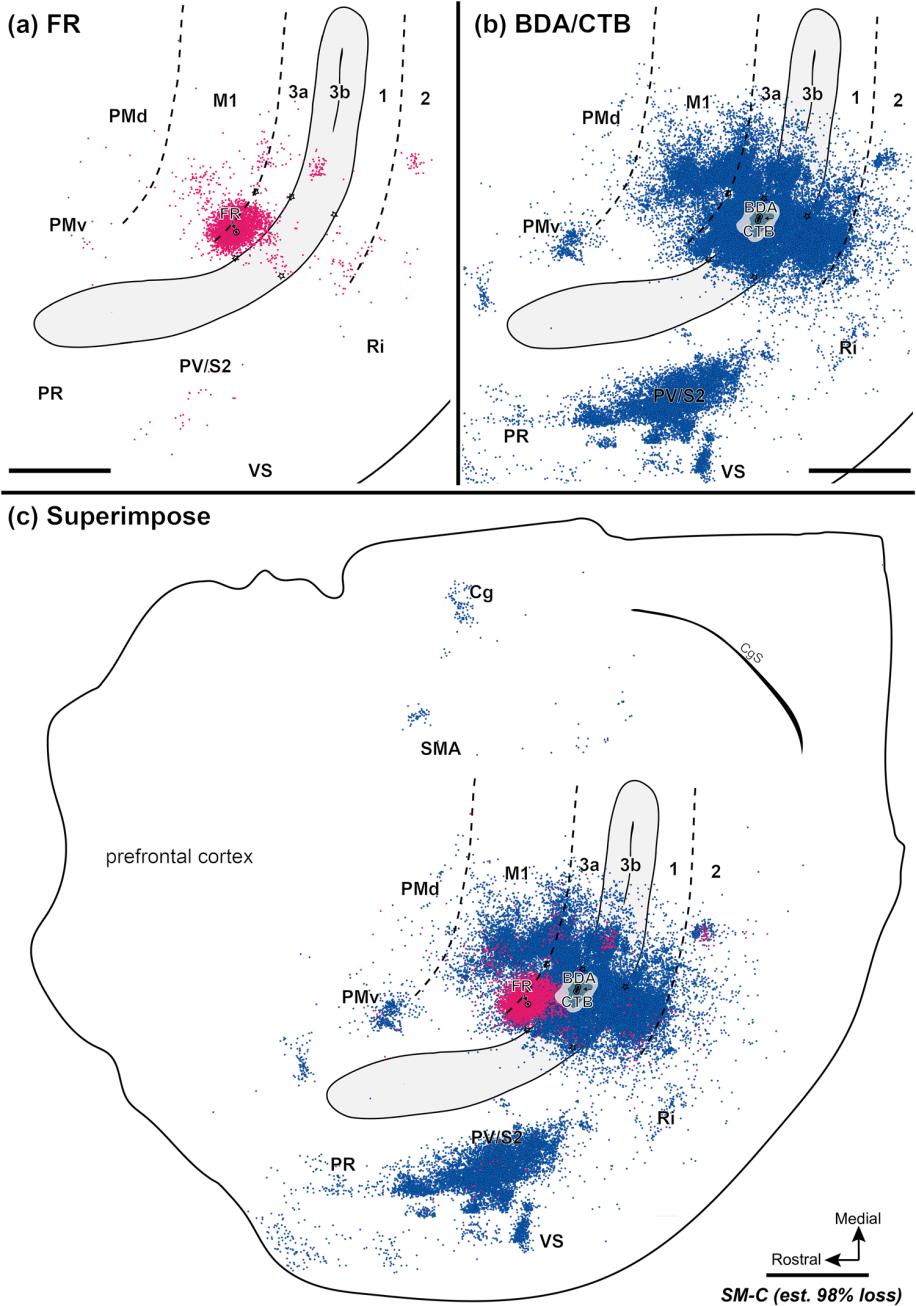
Distribution of labeled neurons from FR injection into the area 3a elbow movement zone (a, red) and BDA and CTB injections into the 3b elbow movement zone (b, blue) in the cortex contralateral to the dorsal column lesion for case SM‐C. (c) Neurons labeled from the 3a injection are largely confined in the vicinity of the injection site over the hand region of 3a and the caudal part of M1 across the 3a–M1 border; less dense clusters of FR‐labeled neurons scattered in the rest of M1, 3a, 3b, 1, and 2. Very few labeled neurons are found in S2, PV, Ri, PR, and PMv. Neurons labeled from the 3b injection are extensively distributed in hand and forelimb regions of areas M1, 3a, 3b, 1, S2, and PV. Abbreviations and conventions as in Figure 7. Scale bars, 5 mm.

In the DCL monkey SM‐C (98% sensory loss from hand), BDA and CTB were injected into an elbow movement region in deprived area 3b. FR was injected into an elbow movement region in deprived area 3a. Neurons at these sites were not responsive to touch on the hand (Figure 3a,b). The BDA and CTB injection core was slightly more rostral than the injection in the control case SM‐R, but was still confined to the hand region of area 3b (Figure 8). The dense uptake zones encroached on the 3a–3b border. Densely packed BDA‐ and CTB‐labeled neurons were distributed across somatosensory areas 3a, 3b, 1, S2, and PV, and motor area M1. Less dense clusters of labeled neurons were in somatosensory area 2, PR, VS, and frontal motor areas PMd, PMv, SMA, and Cg. FR‐labeled neurons were observed in the forelimb regions of area 3a, extending rostrally into M1. Scatterings of labeled neurons were also in the hand regions of somatosensory areas 3b, 1, 2, S2, and PV. A few FR‐labeled neurons were in premotor areas (Figure 8). Thus, the locations of these sparsely labeled connections of the area 3a elbow movement region were similar to those of the more densely labeled connections of the area 3b elbow movement region. The exception to the similarity was the dense labeling in VS after injections into the area 3b elbow movement region that was not apparent after injections in area 3a.

**FIGURE 9:**
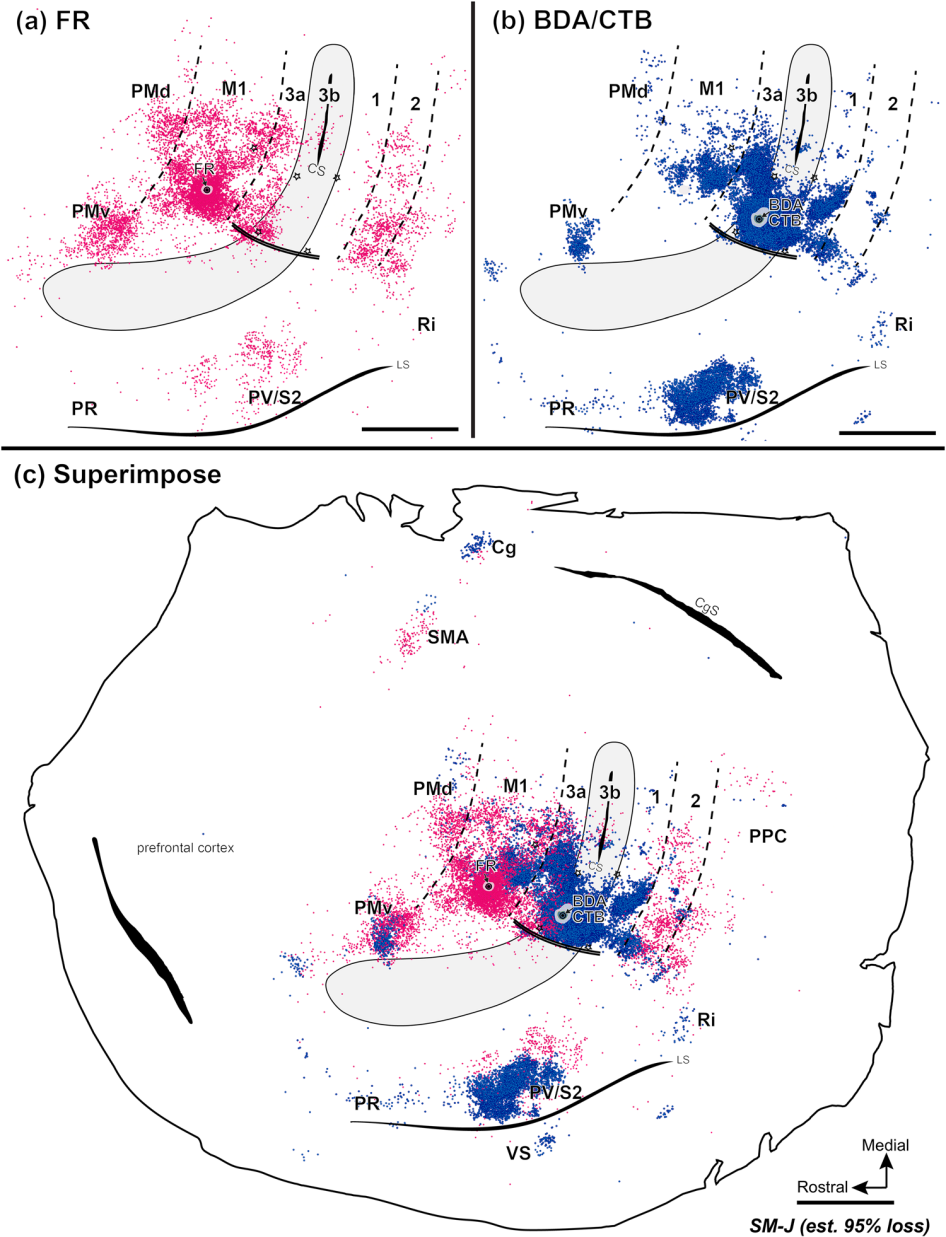
Distribution of labeled neurons from FR injection into the M1 digit and forelimb zone (a, red) and when BDA and CTB mixture was injected into the area 3b digit movement zone (b, blue) in the cortex contralateral to the dorsal column lesion for case SM‐J. (c) Neurons labeled from the M1 injection are extensively distributed in areas M1, 3a, and 2; less dense clusters of labeled neurons are distributed in motor areas PMd, PMv, SMA, Cg, as well as in somatosensory areas S2 and PV. Neurons labeled from the 3b injection are extensively distributed in the hand regions of areas 3a, 3b, 1, S2, and PV, and slightly less densely distributed in M1. Two populations of neurons (blue and red) overlapped in PMv, S2, and PV; with less tight overlaps in SMA and Cg. Abbreviations and conventions as in Figure 7. Scale bars, 5 mm.

In DCL monkey SM‐J (est. 95% sensory loss from hand), a BDA and CTB mixture was injected into the deprived area 3b region, where LT‐ICMS elicited digit movements, and neurons at this site responded to touch on digit 1 (Figure 3c). FR was injected into the M1 territory of concurrent movements of digits, wrist, and elbow (Figure 3d). FR‐labeled neurons appeared to be more widespread mediolaterally within the forelimb region of areas M1, 3a, and 2 (Figure 9a). This may be the substrate related to the abnormal map in the forelimb region of M1 (Figure 3d). Beyond the injection cores and dense uptake zones, FR‐labeled neuron clusters overlapped with BDA‐ and CTB‐labeled neurons in the premotor cortex PMv. However, fewer overlapping clusters of the two populations of labeled neurons were observed in motor areas SMA and Cg and somatosensory areas 2, S2, and PV (Figure 9c).

**FIGURE 10:**
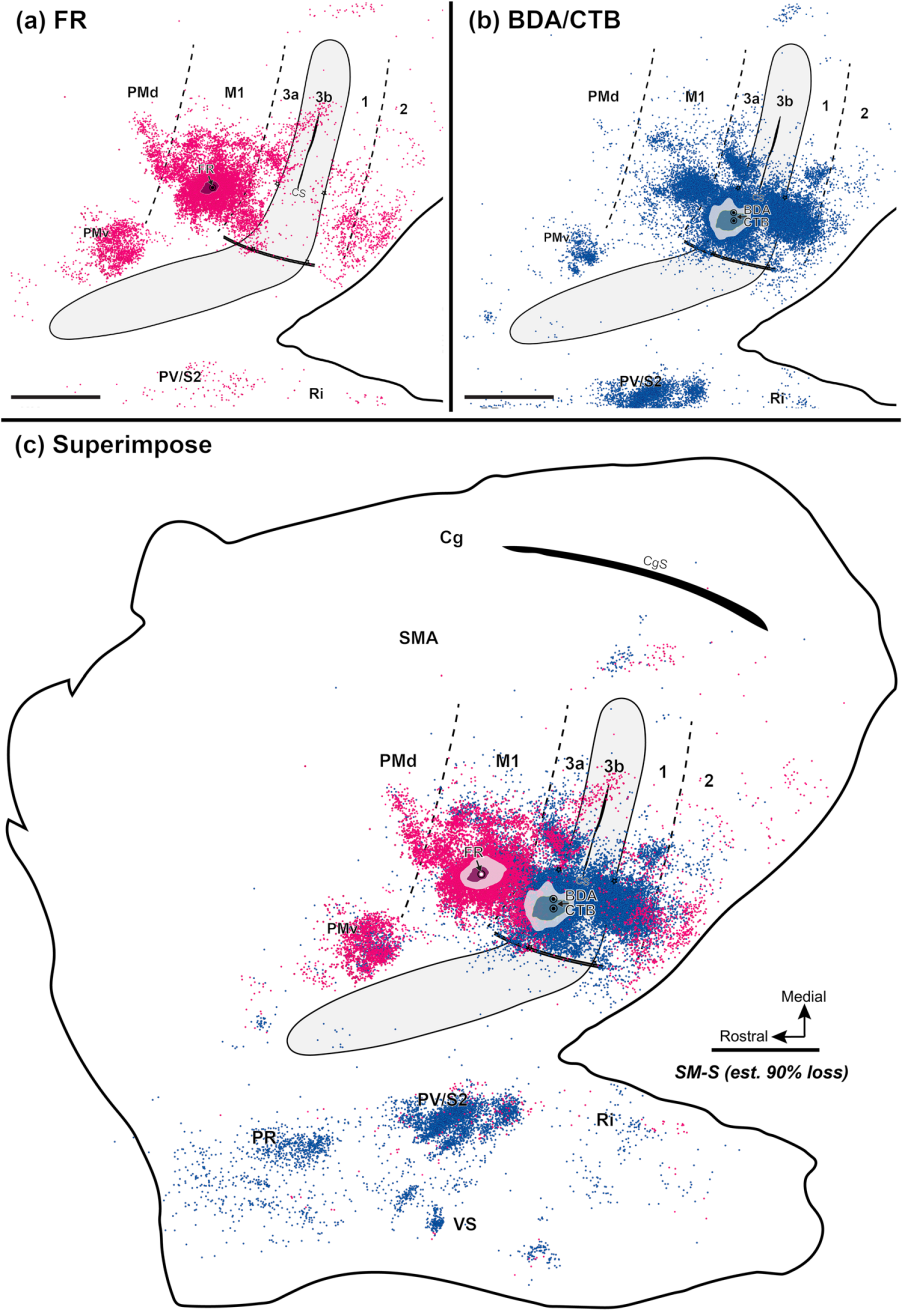
Distribution of labeled neurons following FR injection into the M1 digit and elbow sites (a, red), as well as BDA and CTB injections into area 3b near the 3a–3b border of the digit, wrist, and elbow movement zone (b, blue) in the cortex contralateral to the dorsal column for case SM‐S. (c) Neurons labeled from the M1 injection are extensively distributed across areas M1, 3a, PMd, and PMv; clusters of less densely labeled neurons can be found in areas 2 and the expected hindlimb region of 3b. Neurons labeled from the 3b injection are extensively distributed in the hand regions of areas 3a, 3b, 1, S2, and PV, with a fairly dense population of BDA‐ and CTB‐labeled neurons present in M1, 2, and PR. Abbreviations and conventions as in Figure 7. Scale bars, 5 mm.

In DCL monkey SM‐S (est. 90% sensory loss from hand), BDA and CTB were injected into the hand region of area 3b, where LT‐ICMS evoked movements of the elbow (BDA) and wrist (CTB), with digit movement at a nearby site; and neurons at this site responded to touch on digit 2 (Figure 3e,f). The large BDA and CTB injection core was primarily confined within the hand region of area 3b, where neurons responded to touch on digits, while the uptake zone encroached slightly into the caudal portion of 3a. As shown in Figure 10, beyond the injection cores and dense uptake zones, clusters of the two populations of labeled neurons overlapped in M1 and 3a medial to the injection cores. They overlapped somewhat less in motor areas PMd and PMv. Labeled neurons tended to be more scattered across somatosensory areas 1, 2, S2, and PV. The distribution patterns of labeled neurons were similar to those of monkey SM‐J, except there was no evidence of labeled neurons in SMA and Cg.

#### 3.3.2 Semiquantitative Analysis of Labeled Neuron Distributions

**FIGURE 11:**
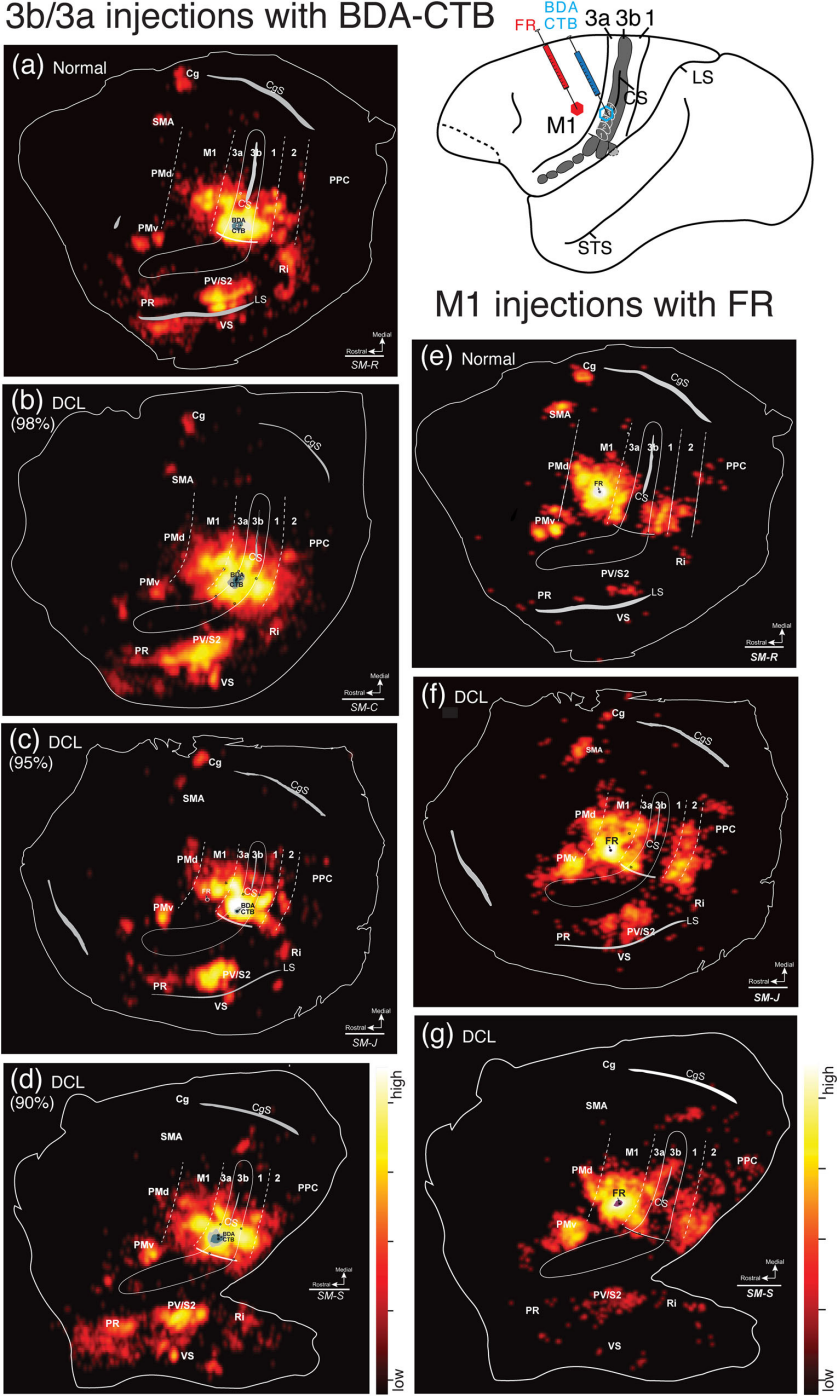
Heatmaps depict labeled cell density. The left column shows labeled cell density after BDA and CTB injections into the hand region of 3b or near the 3a–3b border region in control case SM‐R (a) and lesioned cases of SM‐C (b), SM‐J (c), and SM‐S (d). The injection cores and halos are marked in light blue and gray. The lighter color indicates a higher density of labeled neurons. The right column shows labeled cell density after FR injections into forelimb regions of M1 in control case SM‐R (e) with those of lesioned cases SM‐S (f) and SM‐J (g). The injection cores where the label cannot be counted are indicated in dark red. The lighter color indicates a higher density of labeled neurons. PPC, posterior parietal cortex. Other abbreviations and conventions as in Figure 7. Scale bars, 5 mm.

Heatmaps (Wang et al. 2021) allowed us to compare labeled cell densities from tracer injections in the control monkey (Figure 11a,e) with those in lesioned monkeys (Figure 11b–d and f,g) on the same semiquantitative density scale. The areal distributions and densities of labeled neurons between the control monkey and those of the lesioned monkeys were overall similar, with the following notable exceptions. Widespread labeling occurred, such that the distributions of BDA‐ and CTB‐labeled neurons projecting to 3b or near the border of 3a–3b were slightly more spread in the rostral portion of M1 (Figure 11d) than in the control monkey (Figure 11a). Similarly, the distributions of FR‐labeled neurons projecting to M1 injection sites were slightly more spread out in somatosensory areas S2, PV, and area 2 in monkeys with lesions. Also, after injections in M1 of lesioned monkeys, more sparsely labeled neurons were found in the rostral portion of M1 and premotor cortex (SM‐J and SM‐S; Figure 11f,g). Projections to M1 from SMA and Cg were inconsistently labeled.

Overall, compared to the normal cortex, cells with projections to M1 injection regions appeared to be more densely labeled in higher order somatosensory areas 3a, 2, S2, PV, and VS, and motor areas PMd and PMv in lesioned monkeys. Projections to M1 from area 3b were sparse in the normal cortex, as expected, while the density of labeled projections from parts of area 3b surrounding the deafferented hand territory appeared to be higher in lesioned monkeys. Compared to the normal cortex, neurons with projections to the area 3b hand territory appeared to be higher in density and with more widely spread labeling in the region of areas S2 and PV; and neurons with projections from M1, especially rostral M1, appeared to be higher in density in lesioned monkeys as compared to those of the normal monkey.

#### 3.3.3 Distributions of Labeled Neurons With Forelimb Movement Territories in Area 3b

To determine whether the evoked movement territories in area 3b share specific neuronal connectivity with matching movement regions in M1 or 3a, we superimposed the number of BDA‐ and CTB‐ or FR‐labeled neurons in the hand region of area 3b onto the electrical stimulation maps for all studied cases. Thus, the total number of labeled neurons in area 3b for each evoked movement category was obtained (Table 4). The values were graphed to view the distributions of labeled cells in area 3b localized to evoked movement categories compared to the movement territory injected with the tracers.

**TABLE 4:** Distributions of labeled cells in area 3b from tracer injections into areas 3b and M1.

	Evoked movement	Number	%	Number	%
		BDA/CTB injection into 3b		FR injection into M1	
SM‐R (normal)	Digits	3677	30.3%	3	10.7%
	Wrist	53	0.4%	0	0.0%
	Elbow	1561	12.9%	0	0.0%
	Shoulder	0	0.0%	0	0.0%
	Digit + wrist	124	1.0%	0	0.0%
	Digit + elbow	363	3.0%	0	0.0%
	Wrist + elbow	4	0.0%	0	0.0%
	Wrist + shoulder	23	0.2%	0	0.0%
	Wrist + elbow + shoulder	0	0.0%	0	0.0%
	F/N	625	5.2%	0	0.0%
	No response	5701	47.0%	25	89.3%
	**Total labeled cells in 3b**	**12,131**	**100.0%**	**28**	**100.0%**
		**BDA/CTB injection into 3b**		**FR injection into 3a/M1**	
SM‐C (est. 98% loss from hand)	Digits	0	0.0%	0	0.0%
	Wrist	47	0.4%	0	0.0%
	Elbow	2698	22.2%	34	34.7%
	Shoulder	127	1.0%	0	0.0%
	Digit + wrist	0	0.0%	0	0.0%
	Digit + elbow	149	1.2%	0	0.0%
	Wrist + elbow	0	0.0%	0	0.0%
	Wrist + shoulder	0	0.0%	0	0.0%
	Wrist + elbow + shoulder	0	0.0%	0	0.0%
	F/N	250	2.1%	5	5.1%
	No response	8890	73.1%	59	60.2%
	**Total labeled cells in 3b**	**12,161**	**100.0%**	**98**	**100.0%**
		**BDA/CTB injection into 3b**		**FR injection into M1**	
SM‐J (est. 95% loss from hand)	Digits	4143	30.4%	50	24.4%
	Wrist	740	5.4%	15	7.3%
	Elbow	428	3.1%	2	1.0%
	Shoulder	0	0.0%	0	0.0%
	Digit + wrist	284	2.1%	0	0.0%
	Digit + elbow	139	1.0%	5	2.4%
	Wrist + elbow	0	0.0%	0	0.0%
	Wrist + shoulder	0	0.0%	0	0.0%
	Wrist + elbow + shoulder	103	0.8%	0	0.0%
	F/N	81	0.6%	0	0.0%
	No response	7689	56.5%	133	64.9%
	**Total labeled cells in 3b**	**13,607**	**100.0%**	**205**	**100.0%**
		**BDA/CTB injection into 3a‐3b**		**FR injection into M1**	
SM‐S (est. 90% loss from hand)	Digits	101	1.2%	1	1.1%
	Wrist	465	5.7%	23	26.4%
	Elbow	519	6.4%	2	2.3%
	Shoulder	0	0.0%	0	0.0%
	Digit + wrist	655	8.1%	0	0.0%
	Digit + elbow	67	0.8%	0	0.0%
	Wrist + elbow	189	2.3%	2	2.3%
	Wrist + shoulder	0	0.0%	0	0.0%
	Wrist + elbow + shoulder	0	0.0%	0	0.0%
	F/N	22	0.3%	0	0.0%
	No response	6083	75.1%	59	67.8%
	**Total labeled cells in 3b**	**8101**	**100.0%**	**87**	**100.0%**

Quantitative data listing the number and percentage of labeled neurons in area 3b categorized by body movement.

Abbreviations are consistent with those in Figures 6 and 7.

##### 3.3.3.1 Anatomical and Functional Relationship After BDA and CTB Injected Into the Hand Region of Somatosensory Area 3b

**FIGURE 12:**
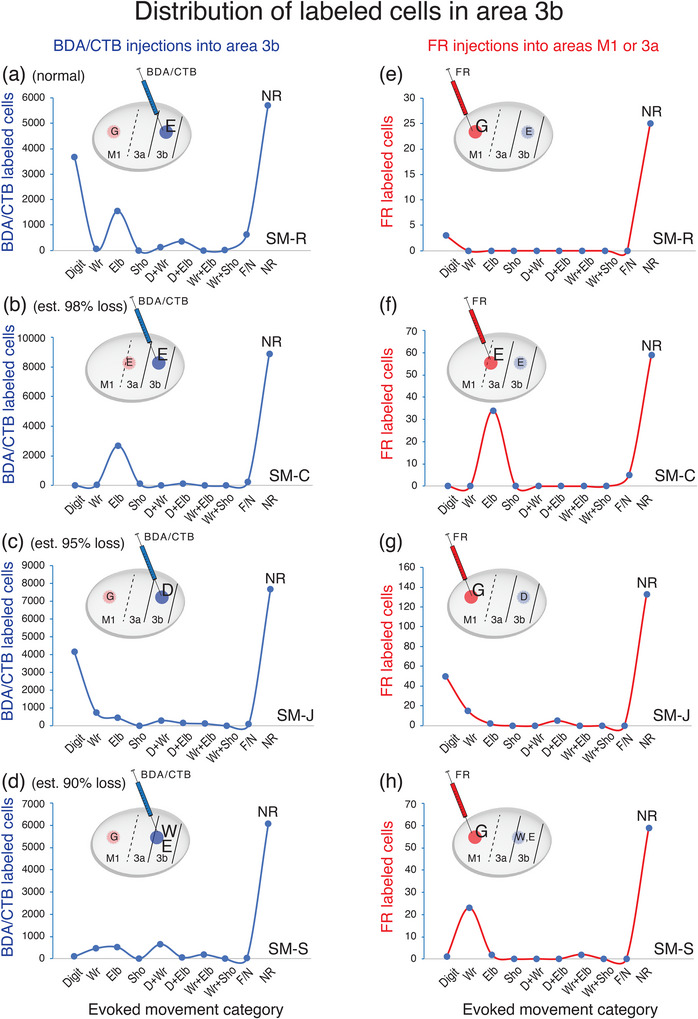
Distribution of labeled neurons within each body movement zone in area 3b after BDA and CTB injections into area 3b (blue) and FR injections into areas M1 or 3a (red) for squirrel monkeys SM‐R, SM‐C, SM‐S, and SM‐J. (a–h) Line graphs depict profiles of labeled neuron numbers in area 3b (*y*‐axis) based on the evoked movements of body parts (*x*‐axis). Schematic drawings with gray ovals in (a–h) indicate the locations of tracer injections, and shown on each blue or red circle are the abbreviations of elicited movements where the tracers were injected. Note that the total number of labeled neurons in area 3b differed drastically in the two sets of plotted tracers, mainly due to two factors. One is that BDA and CTB tracers were directly injected into the hand region of area 3b, where cell counting was made; and the other is that direct connections between areas 3b and M1 are rare. D, digit; D+ Elb, concurrent movements of digits and elbow; DEW, evoked movements of digit, elbow, wrist; D+ Wr, concurrent movements of digits and wrist; E or Elb, elbow; F/N, joint movement or muscle twitch from the face, ear, jaw, neck; G, grasp (digits and forearm supination); N/R, no response to electrical stimulation at 150 µA; Sho, shoulder; Wr, wrist; W, E, wrist or elbow; Wr + Elb, concurrent movements of wrist and elbow; Wr + Sho, concurrent movements of wrist and shoulder.

The distributions of BDA‐ and CTB‐ and FR‐labeled neurons in the hand region of area 3b within different evoked forelimb movement territories are illustrated in line graphs for each case (Figure 12). As shown in Table 4, large numbers of labeled neurons in the forelimb region of area 3b were found outside of the responsive zones determined by LT‐ICMS mapping for all studied cases with or without DCL. Note that the category of “no response” (NR) in the context of matching labeled neurons to evoked movements includes the sites from LT‐ICMS mapping that were listed as NR, as well as regions that were outside of the sites mapped. From injections into area 3b, the percentage of BDA‐ and CTB‐labeled neurons in area 3b outside of the response zones varied among four studied cases, with the lowest percentage found in the control monkey SM‐R (47%, 5701 out of 12,131 labeled cells), and higher percentages found in lesioned monkeys SM‐C (73%, 8890 out of 12,161), SM‐J (57%, 7689 out of 13,607), and SM‐S (75%, 6083 out of 8101; Table 4).

In the control monkey SM‐R, the BDA and CTB injections were placed into the hand region of area 3b where electrical stimulation evoked elbow movement, near digit and face movement sites. Within the evoked movement zones, the densest concentration of labeled neurons was near the injected forelimb movement territories in area 3b. Thirty percent of labeled neurons were found in area 3b digit movement territory (3677 out of 12,131 labeled cells; Table 4 and Figure 12a). The second densest concentration of labeled neurons was found in area 3b elbow movement territory (13%, 1561 out of 12,131; Figure 12a).

In DCL monkey SM‐C, BDA and CTB injected into the evoked elbow movement zone of area 3b resulted in a peak in the distribution of labeled neurons in the 3b elbow movement territory (22%, 2698 out of 12,161 labeled cells, Figure 12b and Table 4), within the hand representation of area 3b. In contrast to the control monkey, much fewer BDA‐ and CTB‐labeled neurons were found in other evoked forelimb territories.

In DCL monkey SM‐J, BDA and CTB injected into the evoked digit movement zone of area 3b resulted in densely labeled neurons in the 3b digit movement territory (30%, 4143 out of 13,607, Figure 1c and Table 4), within the hand representation of area 3b.

In the third lesioned monkey SM‐S, BDA and CTB were injected into the digit region of area 3b where LT‐ICMS evoked elbow and wrist movements, near zones that evoked combinations of movements involving digits resulted in clusters of labeled neurons in multiple territories of evoked movements of wrist, elbow, or concurrent movements of digit and wrist (Figure 12d and Table 4), which matched with the functional map produced by electrical stimulation.

##### 3.3.3.2 Anatomical and Functional Relationship After M1 Injections

Fewer neurons were labeled in area 3b from injections into M1, but the percentage of FR‐labeled neurons outside of the response zones was highest in the control monkey SM‐R (89%, 25 out of 28 labeled cells), and lower in the lesioned monkeys SM‐C (60%, 59 out of 98), SM‐J (65%, 133 out of 205), and SM‐S (68%, 59 out of 87; Table 4).

The total number of labeled neurons in the hand region of area 3b after FR injection into a functionally matched region of M1 was much less compared to cells labeled by tracer injections into the hand region of area 3b. This was consistent with other studies showing that there were few direct projections from 3b to M1 in primates (Jones et al. 1978; Krubitzer and Kaas 1990; Darian‐Smith et al. 1993; Wu and Kaas 2003). As expected in normal cortex, very few neurons from area 3b projected to M1 after FR injections into M1 territory for combined movements, including grasping; however, those found (three out of 28 labeled cells) were located where digit movements were evoked in area 3b (Figure 12e and Table 4). Slightly higher percentages of FR‐labeled neurons (peaks in Figure 12e–h) in area 3b tended to be found in territories with movement categories related to the site where FR was injected in the forelimb region of M1. For instance, in DCL monkey SM‐J where FR was injected into the digit grasp site of M1, 24% of labeled neurons were found in the evoked digit territory, 7% of FR‐labeled neurons in wrist territory, and 2% of labeled neurons in the concurrent movement of digit and elbow (2%) region in 3b (Figure 12f and Table 4). In DCL monkey SM‐C, 35% of FR‐labeled neurons were found in the evoked elbow movement zone in area 3b after FR was injected into the elbow movement site of 3a (Figure 12g and Table 4). In DCL monkey SM‐S, 26% of FR‐labeled neurons were found in the evoked wrist movement region after FR was injected into the grasp site in M1 (Figure 12h and Table 4).

Taken together, areal distributions of labeled neurons by 3a, 3b, and M1 injections in DCL monkeys were overall similar to those of the control monkey and were consistent with those of intact monkeys of previous reports (Stepniewska et al. 1993; Dum and Strick 2005; Gharbawie et al. 2011; Negyessy et al. 2013; Liao et al. 2013, 2016a). Yet, sparse but widespread connections after sensory loss were present based on semiquantitative measurement. The neurons projecting to area 3b tended to be labeled within matching evoked movement regions relative to that of the injection zone rather than dispersed across regions related to evoked movements of other parts of the forelimb.

## 4 Discussion

Our goal was to determine if a major loss of somatosensory inputs to the sensorimotor cortex would alter the LT‐ICMS maps of the movement patterns in the hand cortex in somatosensory areas (3a, 3b, and 1) and primary motor cortex, even after 1 year of postlesion recovery. We report that LT‐ICMS can elicit digit and forelimb movements in the forelimb regions of somatosensory areas 3a, 3b, and 1 in monkeys with and without sensory loss due to DCL. The cortical connections labeled by tracer injections into electrophysiologically defined evoked movement regions in deprived but recovered somatosensory cortex are consistent with those reported in previous studies of area 3b connections in deprived and normal monkeys (e.g., Liao et al. 2013, 2016a) and of M1 connections in normal New World monkeys (Stepniewska et al. 1993; Dum and Strick 2005; Gharbawie et al. 2011). We found that long‐term recoveries from DCL led to increased connections to M1 from somatosensory areas 1, 2, S2, and PV, as well as widespread connections to area 3b, with increased connections between M1 and area 3b. These connections are likely one substrate for the resultant motor response maps in individual monkeys. However, we did not detect robust effects of long‐term DCL on the properties of evoked motor responses in squirrel monkeys, suggesting recovery to nearly normal.

The lack of topography of movement representations in somatosensory areas 3a, 3b, and 1 is in stark contrast to the discrete, orderly arrangement of the neuronal receptive fields that represent the skin surfaces (e.g., Merzenich et al. 1978). However, the evoked movement patterns of M1 in normal New World monkeys lack orderly topographic organization within forelimb territories (Donoghue et al. 1992; Nudo et al. 1992), and interanimal variation is influenced by several factors, including injury‐ or use‐dependent plasticity (e.g., Nudo et al. 1996; Nudo 1999). The ICMS patterns of motor response organization of M1 that we report here align with previous studies of intact squirrel monkeys (Donoghue et al. 1992; Nudo et al. 1992; Wu and Kaas 1999; Dancause et al. 2006; Card and Gharbawie 2020; Plauzt et al. 2023), owl monkeys (Gould et al. 1986), and capuchin monkeys (Mayer et al. 2019). While digit movement patterns varied among lesioned cases, the overall topography of forelimb movement representations in M1 remained consistent after DCL, a result also observed in macaque monkeys with DCL (Qi et al. 2010; Kambi et al. 2011).

### 4.1 Motor Response Map Organization in Sensorimotor Cortex After Sensory Loss From the Hand

Fine motor control of the hand requires integration of tactile and proprioceptive information. Sensory loss can result in disuse of the affected hand, and disuse can cause further sensorimotor impairments (see Taub 2014 for review). Like the impact on the somatosensory map (Jain et al. 2008), the impact on the motor map at the time of the lesion is likely to be great. Note that we did not test arm movements and manual dexterity with specific tasks for these cases. Our postlesion records indicated that they were severely impaired for 1 week of observation. The in‐cage behavior of these monkeys did not differ from that of normal monkeys by the time of cortical mapping. In our previous studies, with similar spinal cord lesion extents as the present cases, animals showed over 90% success rate recovery of performance on a reach and retrieval task 7–12 weeks after the lesion (Qi and Reed et al. 2014, Qi et al. 2021). Thus, the present squirrel monkeys with extensive sensory loss are expected to have recovered their hand use for reaching and grasping by the time of the mapping procedure.

We propose the following interpretation to explain our results of motor response maps after long‐term unilateral DCL. As sensory reactivation occurs and normal or near‐normal reaching and grasping behavior returns, the motor map in the sensory cortex returns. In M1, normal complex movements return with normal thresholds for evoke movements. In S1, the deprived hand territory becomes reactivated by remaining hand inputs and, in part, by inputs that represent the arm in somatosensory and motor maps. The neurons in the deprived somatosensory cortex then require increased current levels to evoke movements. Thus, we propose that the results of considerable cortical reactivation a year after DCL occur after tactile and proprioceptive activation reach levels so that motor control is near normal. Thresholds for evoking movements from the parts of cortex deprived of primary inputs then may be higher than normal for inputs that have new axons and synapses, such as arm inputs in the hand territory.

The anatomical and functional consequences in areas of sensorimotor cortex after sensory loss from DCL are briefly described in terms of (1) direct descending projections altering the excitability of neurons in the spinal cord and (2) corticocortical and thalamocortical connections (for review, see Mohammed and Hollis 2018).

#### 4.1.1 Somatosensory Descending Pathways and Intraspinal Networks

Movement execution in primates relies on the integrity of corticospinal connections. The corticospinal tracts in primates originate from nine or more cortical areas, including primary somatosensory cortex (referred to here as S1), among others (Darian‐Smith et al. 2014; Darian‐Smith and Fisher 2020 for review). Several sensorimotor cortical areas project to the contralateral cuneate nucleus of the brainstem and dorsal horn of the spinal cord in macaque monkeys (Coulter and Jones 1977; Cheema et al. 1985; Bentivoglio and Rustioni 1986), and squirrel monkeys (Wise and Jones 1977). These pathways of different origins have a variety of roles in motor responses (e.g., Darian‐Smith and Fisher 2020, review). It has been unclear whether the corticospinal tracts originating in S1 areas can drive motor responses without acting through cortical connections or cortico‐thalamocortical loops to M1. Among the early studies, Fromm and Evarts (1982) showed that neurons in proprioceptive area 3a of macaque monkeys have a direct role in the control of movement. In macaque monkeys, Witham et al. (2016) found that ICMS in area 3a rarely produced monosynaptic motoneuron responses, but multisynaptic activation was common. Regarding areas 3b and 1, S1 corticospinal tracts may provide feedback that modulates the sensory input at the level of the spinal cord or may be involved in cortical sensory gating (for reviews, Darian‐Smith and Fisher 2020; Kalambogias and Yoshida 2021). However, Bresee et al. (2024) reported that reversible inactivation of M1 did not abolish all ICMS‐driven movements from somatosensory area 1 (34% abolished), indicating that S1 corticospinal tracts can drive motor responses. Given that we report area 1 is the least effective in driving hand movements, we conclude that corticospinal tract projections from areas 3a and 3b also drive motor responses after DCL. Some primates have direct M1 corticospinal tract projections to motoneurons for hand use (for reviews, Lemon 2019; Kalambogias and Yoshida 2021), but it is likely that S1 corticospinal tracts indirectly affect motoneurons for motor responses.

In monkeys, several months after DCL, bilateral sprouting of M1 corticospinal tracts and S1 corticospinal tracts can occur in the cervical spinal cord (Fisher et al. 2020, 2018), and terminations can extend caudally beyond the normal territory from C1 to T4 (Darian‐Smith et al. 2014; Fisher et al. 2018). These spinal cord segments represent sensations and movements of the head, neck, forelimb, and upper trunk. The sprouting axons may serve as anatomical substrates for evoked proximal forelimb movements in the somatosensory hand territory in areas 3a, 3b, and 1. After DCL in macaque monkeys, Fisher et al. (2018) found that the spared primary afferents and S1 corticospinal tract efferents sprouted in an overlapping pattern in the dorsal horn, while larger, faster corticospinal tracts projections were lost, suggesting significantly altered cortical modulation of neurons in the spinal cord. These findings, along with the sprouting of corticospinal tract connections to all of the cervical spinal cord, indicate that changes in connections at multiple levels likely alter the balance of sensorimotor networks (Darian‐Smith et al. 2014; Fisher et al. 2020, 2018).

#### 4.1.2 Sensorimotor Integration Through Corticocortical and Thalamocortical Connections

Neuron recording studies have shown that somatosensory inputs to M1 can modulate motoneuron activity and influence motor responses (Schieber 2001; Rathelot and Strick 2006). ICMS in areas 3b and 1 modulate neuron firing in M1 and premotor areas of awake macaque monkeys (Ruszala et al. 2023) and in M1 of humans (Shelchkova et al. 2023; see Sobinov and Bensmaia 2021 for review). Wannier et al. (1991) showed that neurons in areas 3a, 3b, 1, and 2 of macaque monkeys modulate their firing rates during precision grip in feedback roles rather than serving as primary drivers of movement. Using ICMS combined with intrinsic signal optical imaging in squirrel monkeys, researchers revealed functional connectivity between somatosensory areas 3a, 3b, 1 and 2, and M1 (Friedman et al. 2020), and activation maps from those somatosensory sites corresponded closely with connection patterns revealed with neuroanatomical tracer injections into somatosensory representations of individual digits (Card and Gharbawie 2020).

M1 and premotor areas receive most of their somatosensory inputs from areas 3a, 1, 2, S2, and PV (Rosen and Asanuma 1972; Lemon and Porter 1976; Strick and Preston 1982; Fetz et al. 1980; Pons and Kaas 1986; Qi et al. 2002; Disbrow et al. 2003; Coq et al. 2004; Padberg et al. 2005; Gharbawie et al. 2011). These somatosensory areas have dense interconnections with area 3b (Krubitzer and Kaas 1990; Darian‐Smith et al. 1993; Stepniewska et al. 1993; Fang et al. 2005). Thus, proprioceptive inputs from area 3a reach M1 quickly, and there are several ways that tactile inputs from area 3b may reach M1 to affect motor responses through transsynaptic corticocortical connections.

M1 has few inputs from the ventroposterior nucleus of the thalamus (VP), and those connections reflect the accessibility of cortical motor areas to somatosensory DC pathways as well as sensorimotor cerebellum‐recipient thalamic activity (Sommer 2003, review). Researchers found that neurons in the oral part of the ventroposterior lateral nucleus of the thalamus (VPLo) both receive peripheral input and project to the motor cortex (Horne and Tracey 1979; Lemon and van der Burg 1979); but M1 neurons were unresponsive to peripheral stimuli after chronic DCL (Brinkman et al. 1978; Asanuma et al. 1980). Inactivating M1 reduced ICMS‐evoked movements of area 1 in some sites (34%, Bresee et al. 2024); thus, the motor response maps in somatosensory areas 3a, 3b, and 1 appear to primarily arise from S1 corticospinal tract connections, but also reflect the integration of corticocortical and thalamocortical connections with M1.

DCL at the C5 spinal cord level primarily deprives area 3b of sensory input from the hand. Presumably, the activation of a subset of neurons in higher order somatosensory areas would be strengthened by surviving inputs, likely from the proximal forelimb, due to the lack of competition from digit and hand inputs. Thus, somatosensory inputs to M1 are indirectly compromised by the DCL.

## 5 Conclusions

Our results provide the following conclusions. (1) LT‐ICMS in the forelimb territories of the primary somatosensory cortex (area 3b), as well as areas 3a and 1, reveal motor maps of the hand and forelimb in squirrel monkeys. We suggest that these motor maps exist in these sensory areas in all primates. (2) The motor maps in the somatosensory cortex are not highly impacted by sensory loss after 1 year from the time of injury. (3) The impact of sensory loss on the motor map in the motor cortex M1 is not obvious after long recovery, and the motor map in M1 is less dependent on somatosensory inputs. (4) After long‐term DCL, the anatomical connections between somatosensory areas with each other and with M1 are similar to those in intact monkeys, with the notable difference of sparsely scattered and widely spread connections between M1 and area 3b in addition to connections that largely match the expected representation territories. Our findings of cortical connections of somatosensory and motor cortex that spread widely within those somatosensory areas likely contribute to somatosensory functions and the return of compensated hand use. This step in understanding the roles of somatosensory inputs in movement is important for harnessing the sensorimotor interplay to promote recovery from injury.

## Author Contributions

Hui‐Xin Qi, Chia‐Chi Liao, Jamie L. Reed, and Jon H. Kaas designed research; Hui‐Xin Qi, Chia‐Chi Liao, Jamie L. Reed, Iwona Stepniewska, and Qimeng Wang performed research; Hui‐Xin Qi, Chia‐Chi Liao, and Jamie L. Reed analyzed data; Hui‐Xin Qi, Chia‐Chi Liao, Jamie L. Reed, and Jon H. Kaas wrote the manuscript; Iwona Stepniewska and Qimeng Wang edited the manuscript.

## Conflicts of Interest

The authors declare no conflicts of interest. Dr. Jon H. Kaas is an Editorial Board member of this submitted *JOGR Journal* and the corresponding author of this article. To minimize bias, they were excluded from all editorial decision‐making related to the acceptance of this article for publication.

## Data Availability

The data supporting this study's findings are available from the corresponding author upon reasonable request.

## References

[cne70099-bib-0001] Angelucci, A. , F. Clasca , and M. Sur . 1996. “Anterograde Axonal Tracing With the Subunit B of Cholera Toxin: A Highly Sensitive Immunohistochemical Protocol for Revealing Fine Axonal Morphology in Adult and Neonatal Brains.” Journal of Neuroscience Methods 65: 101–112. 8815303 10.1016/0165-0270(95)00155-7

[cne70099-bib-0002] Asanuma, H. , K. Larsen , and H. Yumiya . 1980. “Peripheral Input Pathways to the Monkey Motor Cortex.” Experimental Brain Research 38: 349–355. 6245903 10.1007/BF00236655

[cne70099-bib-0003] Asanuma, H. , and I. Rosen . 1972. “Functional Role of Afferent Inputs to the Monkey Motor Cortex.” Brain Research 40: 3–5. 4624489 10.1016/0006-8993(72)90098-4

[cne70099-bib-0004] Balaram, P. , T. A. Hackett , and J. H. Kaas . 2013. “Differential Expression of Vesicular Glutamate Transporters 1 and 2 May Identify Distinct Modes of Glutamatergic Transmission in the Macaque Visual System.” Journal of Chemical Neuroanatomy 50–51: 21–38. 10.1016/j.jchemneu.2013.02.007 PMC3695749 23524295

[cne70099-bib-0005] Balaram, P. , and J. H. Kaas . 2014. “Towards a Unified Scheme of Cortical Lamination for Primary Visual Cortex Across Primates: Insights From NeuN and VGLUT2 Immunoreactivity.” Frontiers in Neuroanatomy 8: 81. 25177277 10.3389/fnana.2014.00081 PMC4133926

[cne70099-bib-0006] Baldwin, M. K. , P. Balaram , and J. H. Kaas . 2013. “Projections of the Superior Colliculus to the Pulvinar in Prosimian Galagos (*Otolemur garnettii*) and VGLUT2 Staining of the Visual Pulvinar.” Journal of Comparative Neurology 521: 1664–1682. 23124867 10.1002/cne.23252 PMC3579010

[cne70099-bib-0007] Baldwin, M. K. , D. F. Cooke , and L. Krubitzer . 2017. “Intracortical Microstimulation Maps of Motor, Somatosensory, and Posterior Parietal Cortex in Tree Shrews (*Tupaia belangeri*) Reveal Complex Movement Representations.” Cerebral Cortex 27: 1439–1456. 26759478 10.1093/cercor/bhv329 PMC6075024

[cne70099-bib-0008] Baldwin, M. K. L. , D. F. Cooke , A. B. Goldring , and L. Krubitzer . 2018. “Representations of Fine Digit Movements in Posterior and Anterior Parietal Cortex Revealed Using Long‐Train Intracortical Microstimulation in Macaque Monkeys.” Cerebral Cortex 28: 4244–4263. 29136133 10.1093/cercor/bhx279 PMC6215470

[cne70099-bib-0009] Bentivoglio, M. , and A Rustioni . 1986. “Corticospinal Neurons With Branching Axons to the Dorsal Column Nuclei in the Monkey.” Journal of Comparative Neurology 253: 260–276. 3793994 10.1002/cne.902530212

[cne70099-bib-0010] Bresee, C. S. , D. F. Cooke , A. B. Goldring , M. K. L. Baldwin , C. R. Pineda , and L. A. Krubitzer . 2024. “Reversible Deactivation of Motor Cortex Reveals That Areas in Parietal Cortex Are Differentially Dependent on Motor Cortex for the Generation of Movement.” Journal of Neurophysiology 131: 106–123. 38092416 10.1152/jn.00086.2023 PMC11286310

[cne70099-bib-0011] Brinkman, J. , B. M. Bush , and R Porter . 1978. “Deficient Influence of Peripheral Stimuli on Precentral Neurones in Monkeys With Dorsal Column Lesions.” Journal of Physiology 276: 27–48. 418170 10.1113/jphysiol.1978.sp012218 PMC1282409

[cne70099-bib-0012] Brock, A. A. , R. M. Friedman , R. H. Fan , and A. W. Roe . 2013. “Optical Imaging of Cortical Networks via Intracortical Microstimulation.” Journal of Neurophysiology 110: 2670–2678. 24027103 10.1152/jn.00879.2012 PMC3882772

[cne70099-bib-0013] Burish, M. J. , I. Stepniewska , and J. H. Kaas . 2008. “Microstimulation and Architectonics of Frontoparietal Cortex in Common Marmosets (*Callithrix jacchus*).” Journal of Comparative Neurology 507: 1151–1168. 18175349 10.1002/cne.21596

[cne70099-bib-0014] Capaday, C. 2022. “Motor Cortex Outputs Evoked by Long‐Duration Microstimulation Encode Synergistic Muscle Activation Patterns Not Controlled Movement Trajectories.” Frontiers in Computational Neuroscience 16: 851485. 36062251 10.3389/fncom.2022.851485 PMC9434634

[cne70099-bib-0015] Capaday, C. , C. Ethier , C. Van Vreeswijk , and W. G. Darling . 2013. “On the Functional Organization and Operational Principles of the Motor Cortex.” Frontiers in Neural Circuits 7: 66. 23616749 10.3389/fncir.2013.00066 PMC3629310

[cne70099-bib-0016] Card, N. S. , and O. A. Gharbawie . 2020. “Principles of Intrinsic Motor Cortex Connectivity in Primates.” Journal of Neuroscience 40: 4348–4362. 32327531 10.1523/JNEUROSCI.0003-20.2020 PMC7252484

[cne70099-bib-0017] Cheema, S. , A. Rustioni , and B. L. Whitsel . 1985. “Sensorimotor Cortical Projections to the Primate Cuneate Nucleus.” Journal of Comparative Neurology 240: 196–211. 2414345 10.1002/cne.902400209

[cne70099-bib-0018] Chen, L. M. , H. X. Qi , and J. H. Kaas . 2012. “Dynamic Reorganization of Digit Representations in Somatosensory Cortex of Nonhuman Primates After Spinal Cord Injury.” Journal of Neuroscience 32: 14649–14663. 23077051 10.1523/JNEUROSCI.1841-12.2012 PMC3498942

[cne70099-bib-0019] Coq, J. O. , H. Qi , C. E. Collins , and J. H. Kaas . 2004. “Anatomical and Functional Organization of Somatosensory Areas of the Lateral Fissure of the New World Titi Monkey (*Callicebus moloch*).” Journal of Comparative Neurology 476: 363–387. 15282711 10.1002/cne.20237

[cne70099-bib-0020] Coulter, J. D. , and E. G. Jones . 1977. “Differential Distribution of Corticospinal Projections From Individual Cytoarchitectonic Fields in the Monkey.” Brain Research 129: 335–340. 69470 10.1016/0006-8993(77)90012-9

[cne70099-bib-0021] Dancause, N. , S. Barbay , S. B. Frost , et al. 2006. “Ipsilateral Connections of the Ventral Premotor Cortex in a New World Primate.” Journal of Comparative Neurology 495: 374–390. 16485282 10.1002/cne.20875 PMC2583355

[cne70099-bib-0022] Darian‐Smith, C. , and M. M. Ciferri . 2005. “Loss and Recovery of Voluntary Hand Movements in the Macaque Following a Cervical Dorsal Rhizotomy.” Journal of Comparative Neurology 491: 27–45. 16127695 10.1002/cne.20686

[cne70099-bib-0023] Darian‐Smith, C. , I. Darian‐Smith , K. Burman , and N. Ratcliffe . 1993. “Ipsilateral Cortical Projections to Areas 3a, 3b, and 4 in the Macaque Monkey.” Journal of Comparative Neurology 335: 200–213. 8227514 10.1002/cne.903350205

[cne70099-bib-0024] Darian‐Smith, C. , and K. M. Fisher . 2020. “Plasticity of the Somatosensory System After Injury.” In The Sense, a Comprehensive Reference, 2nd ed., edited by B. Fritzsch , 382–392. Elsevier.

[cne70099-bib-0025] Darian‐Smith, C. , A. Lilak , J. Garner , and K. A. Irvine . 2014. “Corticospinal Sprouting Differs According to Spinal Injury Location and Cortical Origin in macaque Monkeys.” Journal of Neuroscience 34: 12267–12279. 25209269 10.1523/JNEUROSCI.1593-14.2014 PMC4160766

[cne70099-bib-0026] Disbrow, E. , E. Litinas , G. H. Recanzone , J. Padberg , and L. Krubitzer . 2003. “Cortical Connections of the Second Somatosensory Area and the Parietal Ventral Area in Macaque Monkeys.” Journal of Comparative Neurology 462: 382–399. 12811808 10.1002/cne.10731

[cne70099-bib-0027] Donoghue, J. P. , S. Leibovic , and J. N. Sanes . 1992. “Organization of the Forelimb Area in Squirrel Monkey Motor Cortex: Representation of Digit, Wrist, and Elbow Muscles.” Experimental Brain Research 89: 1–19. 1601087 10.1007/BF00228996

[cne70099-bib-0028] Dum, R. P. , and P. L. Strick . 2005. “Frontal Lobe Inputs to the Digit Representations of the Motor Areas on the Lateral Surface of the Hemisphere.” Journal of Neuroscience 25: 1375–1386. 15703391 10.1523/JNEUROSCI.3902-04.2005 PMC6726000

[cne70099-bib-0029] Duque, D. H. , J. M. Racca , I. V. Manzanera Esteve , P. F. Yang , J. C. Gore , and L. M Chen . 2023. “Machine‐Learning‐Based Video Analysis of Grasping Behavior During Recovery From Cervical Spinal Cord Injury.” Behavioural Brain Research 443: 114150. 36216141 10.1016/j.bbr.2022.114150 PMC10733977

[cne70099-bib-0030] Fang, P. C. , I. Stepniewska , and J. H. Kaas . 2005. “Ipsilateral Cortical Connections of Motor, Premotor, Frontal Eye, and Posterior Parietal Fields in a Prosimian Primate, *Otolemur garnetti* .” Journal of Comparative Neurology 490: 305–333. 16082679 10.1002/cne.20665

[cne70099-bib-0031] Fetz, E. E. , D. V. Finocchio , M. A. Baker , and M. J. Soso . 1980. “Sensory and Motor Responses of Precentral Cortex Cells During Comparable Passive and Active Joint Movements.” Journal of Neurophysiology 43: 1070–1089. 6766994 10.1152/jn.1980.43.4.1070

[cne70099-bib-0032] Fisher, K. M. , J. P. Garner , and C. Darian‐Smith . 2020. “Reorganization of the Primate Dorsal Horn in Response to a Deafferentation Lesion Affecting Hand Function.” Journal of Neuroscience 40: 1625–1639. 31959698 10.1523/JNEUROSCI.2330-19.2020 PMC7046332

[cne70099-bib-0033] Fisher, K. M. , A. Lilak , J. Garner , and C Darian‐Smith . 2018. “Extensive Somatosensory and Motor Corticospinal Sprouting Occurs Following a Central Dorsal Column Lesion in Monkeys.” Journal of Comparative Neurology 526: 2373–2387. 30014461 10.1002/cne.24491 PMC6366862

[cne70099-bib-0034] Florence, S. L. , T. A. Hackett , and F. Strata . 2000. “Thalamic and Cortical Contributions to Neural Plasticity After Limb Amputation.” Journal of Neurophysiology 83: 3154–3159. 10805710 10.1152/jn.2000.83.5.3154

[cne70099-bib-0035] Florence, S. L. , H. B. Taub , and J. H. Kaas . 1998. “Large‐Scale Sprouting of Cortical Connections After Peripheral Injury in Adult Macaque Monkeys.” Science 282: 1117–1121. 9804549 10.1126/science.282.5391.1117

[cne70099-bib-0036] Florence, S. L. , J. T. Wall , and J. H. Kaas . 1991. “Central Projections From the Skin of the Hand in Squirrel Monkeys.” Journal of Comparative Neurology 311: 563–578. 1721925 10.1002/cne.903110410

[cne70099-bib-0037] Fogassi, L. , V. Gallese , M. Gentilucci , G. Luppino , M. Matelli , and G. Rizzolatti . 1994. “The Fronto‐Parietal Cortex of the Prosimian Galago: Patterns of Cytochrome Oxidase Activity and Motor Maps.” Behavioural Brain Research 60, no. 1: 91–113. 8185856 10.1016/0166-4328(94)90067-1

[cne70099-bib-0038] Friedman, R. M. , K. A. Morone , O. A. Gharbawie , and A. W. Roe . 2020. “Mapping Mesoscale Cortical Connectivity in Monkey Sensorimotor Cortex With Optical Imaging and Microstimulation.” Journal of Comparative Neurology 528, no. 17: 3095–3107. 10.1002/cne.24918. 32255200 PMC7541397

[cne70099-bib-0039] Fromm, C. , and E. V. Evarts . 1982. “Pyramidal Tract Neurons in Somatosensory Cortex: Central and Peripheral Inputs During Voluntary Movement.” Brain Research 238: 186–191. 6805854 10.1016/0006-8993(82)90781-8

[cne70099-bib-0040] Fujiyama, F. , T. Furuta , and T. Kaneko . 2001. “Immunocytochemical Localization of Candidates for Vesicular Glutamate Transporters in the Rat Cerebral Cortex.” Journal of Comparative Neurology 435: 379–387. 11406819 10.1002/cne.1037

[cne70099-bib-0041] Gharbawie, O. A. , I. Stepniewska , and J. H. Kaas . 2011. “Cortical Connections of Functional Zones in Posterior Parietal Cortex and Frontal Cortex Motor Regions in New World Monkeys.” Cerebral Cortex 21: 1981–2002. 21263034 10.1093/cercor/bhq260 PMC3155600

[cne70099-bib-0042] Gibson, A. R. , D. I. Hansma , J. C. Houk , and F. R. Robinson . 1984. “A Sensitive Low Artifact TMB Procedure for the Demonstration of WGA‐HRP in the CNS.” Brain Research 298: 235–241. 6202368 10.1016/0006-8993(84)91423-9

[cne70099-bib-0043] Gould 3rd, H. J. , C. G. Cusick , T. P. Pons , and J. H Kaas . 1986. “The Relationship of Corpus Callosum Connections to Electrical Stimulation Maps of Motor, Supplementary Motor, and the Frontal Eye Fields in Owl Monkeys.” Journal of Comparative Neurology 247: 297–325. 3722441 10.1002/cne.902470303

[cne70099-bib-0044] Graziano, M. S. , C. S. Taylor , and T Moore . 2002a. “Complex Movements Evoked by Microstimulation of Precentral Cortex.” Neuron 34: 841–851. 12062029 10.1016/s0896-6273(02)00698-0

[cne70099-bib-0045] Graziano, M. S. , C. S. Taylor , T. Moore , and D. F Cooke . 2002b. “The Cortical Control of Movement Revisited.” Neuron 36: 349–362. 12408840 10.1016/s0896-6273(02)01003-6

[cne70099-bib-0046] Hackett, T. A. , and L. A. de la Mothe . 2009. “Regional and Laminar Distribution of the Vesicular Glutamate Transporter, VGluT2, in the Macaque Monkey Auditory Cortex.” Journal of Chemical Neuroanatomy 38: 106–116. 19446630 10.1016/j.jchemneu.2009.05.002 PMC2774764

[cne70099-bib-0047] Halley, A. C. , M. K. L. Baldwin , D. F. Cooke , M. Englund , and L. Krubitzer . 2020. “Distributed Motor Control of Limb Movements in Rat Motor and Somatosensory Cortex: The Sensorimotor Amalgam Revisited.” Cerebral Cortex 30: 6296–6312. 32691053 10.1093/cercor/bhaa186 PMC8248848

[cne70099-bib-0048] Harlow, H. F. , and C. N. Woolsey . 1958. Biological and Biochemical Bases of Behaviour: University of Wisconsin Press.

[cne70099-bib-0049] Horne, M. K. , and D. J. Tracey . 1979. “The Afferents and Projections of the Ventroposterolateral Thalamus in the Monkey.” Experimental Brain Research 36: 129–141. 111956 10.1007/BF00238473

[cne70099-bib-0050] Isa, T. , Y. Ohki , B. Alstermark , L. G. Pettersson , and S. Sasaki . 2007. “Direct and Indirect Cortico‐Motoneuronal Pathways and Control of Hand/Arm Movements.” Physiology (Bethesda, Md) 22: 145–152. 17420305 10.1152/physiol.00045.2006

[cne70099-bib-0051] Jain, N. , K. C. Catania , and J. H. Kaas . 1997. “Deactivation and Reactivation of Somatosensory Cortex After Dorsal Spinal Cord Injury.” Nature 386: 495–498. 9087408 10.1038/386495a0

[cne70099-bib-0052] Jain, N. , S. L. Florence , and J. H. Kaas . 1998. “Reorganization of Somatosensory Cortex After Nerve and Spinal Cord Injury.” News in Physiological Sciences 13: 143–149. 11390778 10.1152/physiologyonline.1998.13.3.143

[cne70099-bib-0053] Jain, N. , H. X. Qi , C. E. Collins , and J. H. Kaas . 2008. “Large‐Scale Reorganization in the Somatosensory Cortex and Thalamus After Sensory Loss in Macaque Monkeys.” Journal of Neuroscience 28: 11042–11060. 18945912 10.1523/JNEUROSCI.2334-08.2008 PMC2613515

[cne70099-bib-0054] Jones, E. G. , J. D. Coulter , and S. H. Hendry . 1978. “Intracortical Connectivity of Architectonic Fields in the Somatic Sensory, Motor and Parietal Cortex of Monkeys.” Journal of Comparative Neurology 181: 291–347. 99458 10.1002/cne.901810206

[cne70099-bib-0055] Kalambogias, J. , and Y. Yoshida . 2021. “Converging Integration Between Ascending Proprioceptive Inputs and the Corticospinal Tract Motor Circuit Underlying Skilled Movement Control.” Current Opinion in Physiology 19: 187–193. 33718693 10.1016/j.cophys.2020.10.007 PMC7949357

[cne70099-bib-0056] Kambi, N. , S. Tandon , H. Mohammed , L. Lazar , and N. Jain . 2011. “Reorganization of the Primary Motor Cortex of Adult Macaque Monkeys After Sensory Loss Resulting From Partial Spinal Cord Injuries.” Journal of Neuroscience 31: 3696–3707. 21389224 10.1523/JNEUROSCI.5187-10.2011 PMC3079898

[cne70099-bib-0057] Kaneko, T. , and F. Fujiyama . 2002. “Complementary Distribution of Vesicular Glutamate Transporters in the Central Nervous System.” Neuroscience Research 42: 243–250. 11985876 10.1016/s0168-0102(02)00009-3

[cne70099-bib-0058] Krubitzer, L. A. , and J. H. Kaas . 1990. “The Organization and Connections of Somatosensory Cortex in Marmosets.” Journal of Neuroscience 10: 952–974. 2108231 10.1523/JNEUROSCI.10-03-00952.1990 PMC6570129

[cne70099-bib-0059] Kumaravelu, K. , J. Sombeck , L. E. Miller , S. J. Bensmaia , and W. M. Grill . 2022. “Stoney Vs. Histed: Quantifying the Spatial Effects of Intracortical Microstimulation.” Brain Stimulation 15: 141–151. 34861412 10.1016/j.brs.2021.11.015 PMC8816873

[cne70099-bib-0060] Lemon, R. 2019. “Recent Advances in Our Understanding of the Primate Corticospinal System.” F1000Research 8: F1000. 10.12688/f1000research.17445.1 PMC6415323 30906528

[cne70099-bib-0061] Lemon, R. N. , and R. J. Morecraft . 2023. “The Evidence Against Somatotopic Organization of Function in the Primate Corticospinal Tract.” Brain 146: 1791–1803. 36575147 10.1093/brain/awac496 PMC10411942

[cne70099-bib-0062] Lemon, R. N. , and R. Porter . 1976. “Afferent Input to Movement‐Related Precentral Neurones in Conscious Monkeys.” Proceedings of the Royal Society of London Series B: Biological Sciences 194: 313–339. 11491 10.1098/rspb.1976.0082

[cne70099-bib-0063] Lemon, R. N. , and J. van der Burg . 1979. “Short‐Latency Peripheral Inputs to Thalamic Neurones Projecting to the Motor Cortex in the Monkey.” Experimental Brain Research 36: 445–462. 113234 10.1007/BF00238515

[cne70099-bib-0064] Liao, C. C. , G. E. DiCarlo , O. A. Gharbawie , H. X. Qi , and J. H. Kaas . 2015. “Spinal Cord Neuron Inputs to the Cuneate Nucleus That Partially Survive Dorsal Column Lesions: a Pathway That Could Contribute to Recovery After Spinal Cord Injury.” Journal of Comparative Neurology 523: 2138–2160. 25845707 10.1002/cne.23783 PMC4575617

[cne70099-bib-0065] Liao, C. C. , O. A. Gharbawie , H. Qi , and J. H. Kaas . 2013. “Cortical Connections to Single Digit Representations in Area 3b of Somatosensory Cortex in Squirrel Monkeys and Prosimian Galagos.” Journal of Comparative Neurology 521: 3768–3790. 23749740 10.1002/cne.23377 PMC4000754

[cne70099-bib-0066] Liao, C. C. , H. X. Qi , J. L. Reed , D. J. Miller , and J. H. Kaas . 2016b. “Congenital Foot Deformation Alters the Topographic Organization in the Primate Somatosensory System.” Brain Structure and Function 221: 383–406. 25326245 10.1007/s00429-014-0913-7 PMC4446245

[cne70099-bib-0067] Liao, C. C. , J. L. Reed , J. H. Kaas , and H. X. Qi . 2016a. “Intracortical Connections Are Altered After Long‐Standing Deprivation of Dorsal Column Inputs in the Hand Region of Area 3b in Squirrel Monkeys.” Journal of Comparative Neurology 524: 1494–1526. 26519356 10.1002/cne.23921 PMC4783257

[cne70099-bib-0068] Liao, C. C. , J. L. Reed , H. X. Qi , E. K. Sawyer , and J. H. Kaas . 2018. “Second‐Order Spinal Cord Pathway Contributes to Cortical Responses After Long Recoveries From Dorsal Column Injury in Squirrel Monkeys.” PNAS 115: 4258–4263. 29610299 10.1073/pnas.1718826115 PMC5910841

[cne70099-bib-0069] Maier, M. A. , M. Illert , P. A. Kirkwood , J. Nielsen , and R. N. Lemon . 1998. “Does a C3‐C4 Propriospinal System Transmit Corticospinal Excitation in the Primate? An Investigation in the Macaque Monkey.” Journal of Physiology 511, no. pt 1: 191–212. 9679174 10.1111/j.1469-7793.1998.191bi.x PMC2231097

[cne70099-bib-0070] Mayer, A. , M. K. L. Baldwin , D. F. Cooke , et al. 2019. “The Multiple Representations of Complex Digit Movements in Primary Motor Cortex Form the Building Blocks for Complex Grip Types in Capuchin Monkeys.” Journal of Neuroscience 39: 6684–6695. 31235643 10.1523/JNEUROSCI.0556-19.2019 PMC6703879

[cne70099-bib-0071] Merzenich, M. M. , J. H. Kaas , M. Sur , and C. S. Lin . 1978. “Double Representation of the Body Surface Within Cytoarchitectonic Areas 3b and 1 in "SI" in the Owl Monkey (*Aotus trivirgatus*).” Journal of Comparative Neurology 181: 41–73. 98537 10.1002/cne.901810104

[cne70099-bib-0072] Merzenich, M. M. , J. H. Kaas , J. Wall , R. J. Nelson , M. Sur , and D. Felleman . 1983. “Topographic Reorganization of Somatosensory Cortical Areas 3b and 1 in Adult Monkeys Following Restricted Deafferentation.” Neuroscience 8: 33–55. 6835522 10.1016/0306-4522(83)90024-6

[cne70099-bib-0073] Merzenich, M. M. , R. J. Nelson , J. H. Kaas , et al. 1987. “Variability in Hand Surface Representations in Areas 3b and 1 in Adult Owl and Squirrel Monkeys.” Journal of Comparative Neurology 258: 281–296. 3584541 10.1002/cne.902580208

[cne70099-bib-0074] Mohammed, H. , and E. R. Hollis 2nd . 2018. “Cortical Reorganization of Sensorimotor Systems and the Role of Intracortical Circuits after Spinal Cord Injury.” Neurotherapeutics 15: 588–603. 29882081 10.1007/s13311-018-0638-z PMC6095783

[cne70099-bib-0075] Nahmani, M. , and A. Erisir . 2005. “VGluT2 Immunochemistry Identifies Thalamocortical Terminals in Layer 4 of Adult and Developing Visual Cortex.” Journal of Comparative Neurology 484: 458–473. 15770654 10.1002/cne.20505

[cne70099-bib-0076] Nakajima, K. , M. A. Maier , P. A. Kirkwood , and R. N. Lemon . 2000. “Striking Differences in Transmission of Corticospinal Excitation to Upper Limb Motoneurons in Two Primate Species.” Journal of Neurophysiology 84: 698–709. 10938297 10.1152/jn.2000.84.2.698

[cne70099-bib-0077] Negyessy, L. , E. Palfi , M. Ashaber , et al. 2013. “Intrinsic Horizontal Connections Process Global Tactile Features in the Primary Somatosensory Cortex: Neuroanatomical Evidence.” Journal of Comparative Neurology 521: 2798–2817. 23436325 10.1002/cne.23317 PMC4157923

[cne70099-bib-0078] Nudo, R. J. 1999. “Recovery After Damage to Motor Cortical Areas.” Current Opinion in Neurobiology 9: 740–747. 10607636 10.1016/s0959-4388(99)00027-6

[cne70099-bib-0079] Nudo, R. J. , W. M. Jenkins , M. M. Merzenich , T. Prejean , and R. Grenda . 1992. “Neurophysiological Correlates of Hand Preference in Primary Motor Cortex of Adult Squirrel Monkeys.” Journal of Neuroscience 12: 2918–2947. 1494940 10.1523/JNEUROSCI.12-08-02918.1992 PMC6575643

[cne70099-bib-0080] Nudo, R. J. , G. W. Milliken , W. M. Jenkins , and M. M. Merzenich . 1996. “Use‐Dependent Alterations of Movement Representations in Primary Motor Cortex of Adult Squirrel Monkeys.” Journal of Neuroscience 16: 785–807. 8551360 10.1523/JNEUROSCI.16-02-00785.1996 PMC6578638

[cne70099-bib-0081] Padberg, J. , E. Disbrow , and L. Krubitzer . 2005. “The Organization and Connections of Anterior and Posterior Parietal Cortex in Titi Monkeys: Do New World Monkeys Have an Area 2?” Cerebral Cortex 15: 1938–1963. 15758196 10.1093/cercor/bhi071

[cne70099-bib-0082] Plautz, E. J. , S. Barbay , S. B. Frost , et al. 2023. “Spared Premotor Areas Undergo Rapid Nonlinear Changes in Functional Organization Following a Focal Ischemic Infarct in Primary Motor Cortex of Squirrel Monkeys.” Journal of Neuroscience 43: 2021–2032. 36788028 10.1523/JNEUROSCI.1452-22.2023 PMC10027035

[cne70099-bib-0083] Pons, T. P. , and J. H. Kaas . 1986. “Corticocortical Connections of Area 2 of Somatosensory Cortex in Macaque Monkeys: A Correlative Anatomical and Electrophysiological Study.” Journal of Comparative Neurology 248: 313–335. 3722460 10.1002/cne.902480303

[cne70099-bib-0084] Qi, H. X. , L. M. Chen , and J. H. Kaas . 2011a. “Reorganization of Somatosensory Cortical Areas 3b and 1 After Unilateral Section of Dorsal Columns of the Spinal Cord in Squirrel Monkeys.” Journal of Neuroscience 31: 13662–13675. 21940457 10.1523/JNEUROSCI.2366-11.2011 PMC3183096

[cne70099-bib-0085] Qi, H. X. , O. A. Gharbawie , P. Wong , and J. H. Kaas . 2011b. “Cell‐Poor Septa Separate Representations of Digits in the Ventroposterior Nucleus of the Thalamus in Monkeys and Prosimian Galagos.” Journal of Comparative Neurology 519: 738–758. 21246552 10.1002/cne.22545 PMC3695620

[cne70099-bib-0086] Qi, H. X. , O. A. Gharbawie , K. W. Wynne , and J. H. Kaas . 2013. “Impairment and Recovery of Hand Use After Unilateral Section of the Dorsal Columns of the Spinal Cord in Squirrel Monkeys.” Behavioural Brain Research 252: 363–376. 23747607 10.1016/j.bbr.2013.05.058 PMC3755749

[cne70099-bib-0087] Qi, H. X. , N. Jain , C. E. Collins , D. C. Lyon , and J. H. Kaas . 2010. “Functional Organization of Motor Cortex of Adult Macaque Monkeys Is Altered by Sensory Loss in Infancy.” PNAS 107: 3192–3197. 20133738 10.1073/pnas.0914962107 PMC2840311

[cne70099-bib-0088] Qi, H. X. , and J. H. Kaas . 2006. “Organization of Primary Afferent Projections to the Gracile Nucleus of the Dorsal Column System of Primates.” Journal of Comparative Neurology 499: 183–217. 16977626 10.1002/cne.21061

[cne70099-bib-0089] Qi, H. X. , D. C. Lyon , and J. H. Kaas . 2002. “Cortical and Thalamic Connections of the Parietal Ventral Somatosensory Area in Marmoset Monkeys (*Callithrix jacchus*).” Journal of Comparative Neurology 443: 168–182. 11793354 10.1002/cne.10113

[cne70099-bib-0090] Qi, H. X. , J. L. Reed , F. Wang , et al. 2021. “Longitudinal fMRI Measures of Cortical Reactivation and Hand Use With and Without Training After Sensory Loss in Primates.” Neuroimage 236: 118026. 33930537 10.1016/j.neuroimage.2021.118026 PMC8409436

[cne70099-bib-0091] Qi, H. X. , F. Wang , C. C. Liao , et al. 2016. “Spatiotemporal Trajectories of Reactivation of Somatosensory Cortex by Direct and Secondary Pathways After Dorsal Column Lesions in Squirrel Monkeys.” Neuroimage 142: 431–453. 27523450 10.1016/j.neuroimage.2016.08.015 PMC5159255

[cne70099-bib-0092] Rathelot, J. A. , and P. L. Strick . 2006. “Muscle Representation in the Macaque Motor Cortex: An Anatomical Perspective.” PNAS 103: 8257–8262. 16702556 10.1073/pnas.0602933103 PMC1461407

[cne70099-bib-0093] Rosen, I. , and H. Asanuma . 1972. “Peripheral Afferent Inputs to the Forelimb Area of the Monkey Motor Cortex: Input‐Output Relations.” Experimental Brain Research 14: 257–273. 4626361 10.1007/BF00816162

[cne70099-bib-0094] Ruszala, B. , K. A. Mazurek , and M. H. Schieber . 2023. “Somatosensory Cortex Microstimulation Modulates Primary Motor and Ventral Premotor Cortex Neurons With Extensive Spatial Convergence and Divergence.” *BioRxiv*: 2023.08.05.552025.

[cne70099-bib-0095] Sato, K. C. , and J. Tanji . 1989. “Digit‐Muscle Responses Evoked From Multiple Intracortical Foci in Monkey Precentral Motor Cortex.” Journal of Neurophysiology 62: 959–970. 2681562 10.1152/jn.1989.62.4.959

[cne70099-bib-0096] Schieber, M. H. 2001. “Constraints on Somatotopic Organization in the Primary Motor Cortex.” Journal of Neurophysiology 86: 2125–2143. 11698506 10.1152/jn.2001.86.5.2125

[cne70099-bib-0097] Shelchkova, N. D. , J. E. Downey , C. M. Greenspon , et al. 2023. “Microstimulation of Human Somatosensory Cortex Evokes Task‐Dependent, Spatially Patterned Responses in Motor Cortex.” Nature Communications 14, no. 1: 7270. 10.1038/s41467-023-43140-2. PMC10638421 37949923

[cne70099-bib-0098] Sobinov, A. R. , and S. J. Bensmaia . 2021. “The Neural Mechanisms of Manual Dexterity.” Nature Reviews Neuroscience 22, no. 12: 741–757. 10.1038/s41583-021-00528-7. 34711956 PMC9169115

[cne70099-bib-0099] Stepniewska, I. , C. E. Collins , and J. H. Kaas . 2005. “Reappraisal of DL/V4 Boundaries Based on Connectivity Patterns of Dorsolateral Visual Cortex in Macaques.” Cerebral Cortex 15: 809–822. 15459077 10.1093/cercor/bhh182

[cne70099-bib-0100] Stepniewska, I. , O. A. Gharbawie , M. J. Burish , and J. H. Kaas . 2014. “Effects of Muscimol Inactivations of Functional Domains in Motor, Premotor, and Posterior Parietal Cortex on Complex Movements Evoked by Electrical Stimulation.” Journal of Neurophysiology 111: 1100–1119. 24353298 10.1152/jn.00491.2013 PMC3949230

[cne70099-bib-0101] Stepniewska, I. , T. M. Preuss , and J. H. Kaas . 1993. “Architectonics, Somatotopic Organization, and Ipsilateral Cortical Connections of the Primary Motor Area (M1) of Owl Monkeys.” Journal of Comparative Neurology 330: 238–271. 7684050 10.1002/cne.903300207

[cne70099-bib-0102] Strick, P. L. 2002. “Stimulating Research on Motor Cortex.” Nature Neuroscience 5: 714–715. 12149622 10.1038/nn0802-714

[cne70099-bib-0103] Strick, P. L. , and J. B. Preston . 1982. “Two Representations of the Hand in Area 4 of a Primate. II. Somatosensory Input Organization.” Journal of Neurophysiology 48: 150–159. 7119842 10.1152/jn.1982.48.1.150

[cne70099-bib-0104] Sur, M. , R. J. Nelson , and J. H. Kaas . 1982. “Representations of the Body Surface in Cortical Areas 3b and 1 of Squirrel Monkeys: Comparisons With Other Primates.” Journal of Comparative Neurology 211: 177–192. 7174889 10.1002/cne.902110207

[cne70099-bib-0105] Takahata, T. , N. B. Patel , P. Balaram , Y. M. Chino , and J. H. Kaas . 2018. “Long‐Term Histological Changes in the Macaque Primary Visual Cortex and the Lateral Geniculate Nucleus After Monocular Deprivation Produced by Early Restricted Retinal Lesions and Diffuser Induced Form Deprivation.” Journal of Comparative Neurology 526: 2955–2972. 30004587 10.1002/cne.24494 PMC6283693

[cne70099-bib-0106] Taub, E. 2014. “Foreword for Neuroplasticity and Neurorehabilitation.” Frontiers in Human Neuroscience 8: 544. 25104931 10.3389/fnhum.2014.00544 PMC4109562

[cne70099-bib-0107] Taylor, C. S. , and C. G. Gross . 2003. “Twitches Versus Movements: A Story of Motor Cortex.” Neuroscientist 9: 332–342. 14580118 10.1177/1073858403257037

[cne70099-bib-0108] Wang, Q. , C. C. Liao , I. Stepniewska , M. Gabi , and J. H. Kaas . 2021. “Cortical Connections of the Functional Domain for Climbing or Running in Posterior Parietal Cortex of Galagos.” Journal of Comparative Neurology 529: 2789–2812. 33550608 10.1002/cne.25123 PMC9885969

[cne70099-bib-0109] Wannier, T. M. , M. A. Maier , and M. C. Hepp‐Reymond . 1991. “Contrasting Properties of Monkey Somatosensory and Motor Cortex Neurons Activated During the Control of Force in Precision Grip.” Journal of Neurophysiology 65: 572–589. 2051196 10.1152/jn.1991.65.3.572

[cne70099-bib-0110] Wise, S. P. , and E. G. Jones . 1977. “Cells of Origin and Terminal Distribution of Descending Projections of the Rat Somatic Sensory Cortex.” Journal of Comparative Neurology 175: 129–157. 408380 10.1002/cne.901750202

[cne70099-bib-0111] Witham, C. L. , K. M. Fisher , S. A. Edgley , and S. N. Baker . 2016. “Corticospinal Inputs to Primate Motoneurons Innervating the Forelimb From Two Divisions of Primary Motor Cortex and Area 3a.” Journal of Neuroscience 36: 2605–2616. 26937002 10.1523/JNEUROSCI.4055-15.2016 PMC4879208

[cne70099-bib-0112] Wong, P. , and J. H. Kaas . 2008. “Architectonic Subdivisions of Neocortex in the Gray Squirrel (*Sciurus carolinensis*).” Anatomical Record (Hoboken) 291: 1301–1333. 10.1002/ar.20758 PMC2908424 18780299

[cne70099-bib-0113] Wong, P. , and J. H. Kaas . 2010. “Architectonic Subdivisions of Neocortex in the Galago (*Otolemur garnetti*).” Anatomical Record (Hoboken) 293: 1033–1069. 10.1002/ar.21109 PMC3066689 20201060

[cne70099-bib-0114] Wong‐Riley, M. 1979. “Changes in the Visual System of Monocularly Sutured or Enucleated Cats Demonstrable With Cytochrome Oxidase Histochemistry.” Brain Research 171: 11–28. 223730 10.1016/0006-8993(79)90728-5

[cne70099-bib-0115] Wu, C. W. , and J. H. Kaas . 1999. “Reorganization in Primary Motor Cortex of Primates With Long‐Standing Therapeutic Amputations.” Journal of Neuroscience 19: 7679–7697. 10460274 10.1523/JNEUROSCI.19-17-07679.1999 PMC6782533

[cne70099-bib-0116] Wu, C. W. , and J. H. Kaas . 2003. “Somatosensory Cortex of Prosimian Galagos: Physiological Recording, Cytoarchitecture, and Corticocortical Connections of Anterior Parietal Cortex and Cortex of the Lateral Sulcus.” Journal of Comparative Neurology 457: 263–292. 12541310 10.1002/cne.10542

[cne70099-bib-0117] Sommer, M. A. 2003. “The role of the thalamus in motor control.” Current Opinion in Neurobiology. 3, no. 6: 663–670. 10.1016/j.conb.2003.10.014. 14662366

[cne70099-bib-0118] Turner, E. C. , M. Gabi , C. C. Liao , and J. H. Kaas . 2020. “The postnatal development of MT, V1, LGN, pulvinar and SC in prosimian galagos (Otolemur garnettii).” Journal of Comparative Neurology. 528, no. 17: 30753094. 10.1002/cne.24885. Epub 2020 Feb 24. PMID: 32067231; PMCID: PMC11495416. PMC11495416 32067231

[cne70099-bib-0119] Qi, H. X. , J. L. Reed , O. A. Gharbawie , M. J. Burish , and J. H Kaas . 2014. “Cortical neuron response properties are related to lesion extent and behavioral recovery After sensory loss From spinal cord injury in monkeys.” Journal of Neuroscience. 34, no. 12: 4345–4363. 10.1523/JNEUROSCI.4954-13.2014. PMID: 24647955; PMCID: PMC3960473. 24647955 PMC3960473

